# Supplementary material for: Nowcasting reported covid-19 hospitalizations using de-identified, aggregated medical insurance claims data
Source: PLoS Comput Biol. 2025 Feb 18;21(2):e1012717. doi: 10.1371/journal.pcbi.1012717 (PMC11841917; doi:10.1371/journal.pcbi.1012717)
Supplement: S1 Appendix — (PDF) [file pcbi.1012717.s001.pdf]

S1 Appendix

A More details on claims signals

A.1 Coverage of claims signals

We investigate the coverage of our claims data by comparing the counts of hospitalizations from inpatient claims to the HHS reported counts, over our entire analysis period (November 1, 2020 to August 31, 2023). Fig S1 plots the sum of COVID-19 hospitalizations from these sources, broken down to individual states. Fig S2 plots the percentage of COVID-19 hospitalizations recorded by inpatient claims over the same time period.

A.2 ICD-10 codes for claims signals

For the outpatient signal, any claim that has a primary ICD-10 code of U07.1, B97.21, or B97.29 is counted as confirmed COVID. For the inpatient signal, any claim with a primary ICD-10 code of U07.1, U07.2, B97.29, J12.81, Z03.818, B34.2, or J12.89 is counted as COVID-associated.

These definitions are consistent with the definitions of analogous outpatient and inpatient signals in the Delphi Epidata API. However, the signals that we use in our analysis differ from those in the Delphi Epidata API, at the time that this paper was written, in a way that relates to smoothing: the signals in the API are smoothed with a more sophisticated smoothing approach (which also explicitly adjusts for weekday/weekend differences), whereas the signals in this paper are smoothed via 7-day pooling (which implicitly accounts for weekday/weekend differences).

B Scenario 1: further investigation for NY

We present further analysis as to why state-level model for NY produces negative nowcasts over a portion of the month of June 2021, in scenario 1 (recall Fig 5 in the main paper). Fig S3 displays two versions of the outpatient signal over the month of May 2021, corresponding to issue dates of June 7 and June 8. We can see that a systematic upward revision of all signal values occurs on June 8. The nowcasting model, fit by training on data through the end of May (which sees the revision on June 8), places a sizeable negative weight on the largest lag of the outpatient feature; once the upward revision occurs on June 8, the nowcasts on that and subsequent days display a significant downward trend. This is confirmed by Fig S4, where we decompose the nowcasts made in June into the contributions from each feature (simply the coefficient times the feature value). After June 7, we see that `out_20`, the largest lag of the outpatient figure, is responsible for driving the downward trend.

C Scenario 1: full set of backcasts

Figs S5–S21 display backcasts at lag 0 (i.e., nowcasts), 5, and 10, for all 50 US states and DC, made by the mixed model in scenario 1, the monthly-update period. The format follows Fig 4 in the main paper.

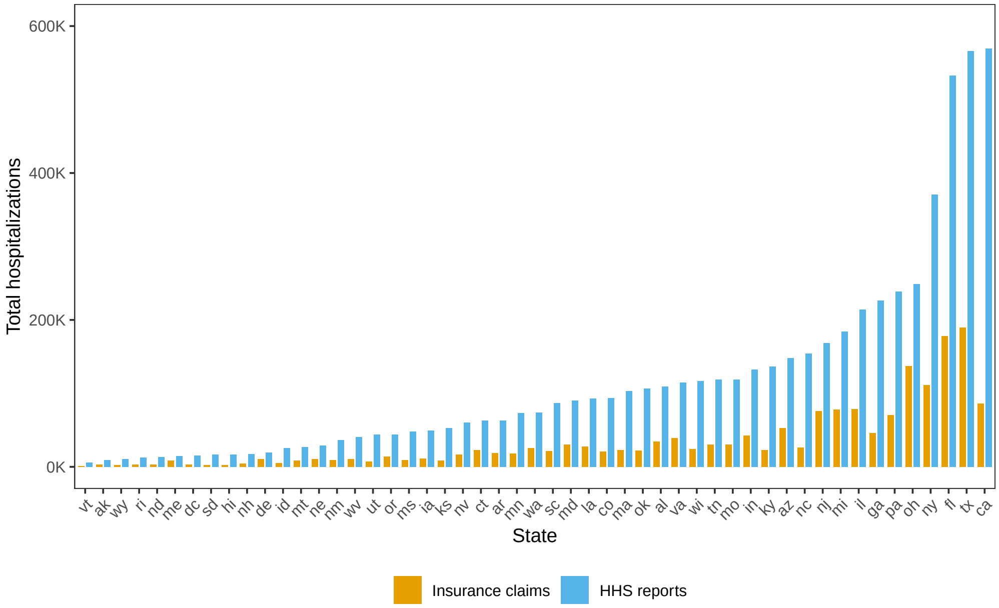

Fig S1. Total number of COVID-19 hospitalizations from official reports and inpatient claims.

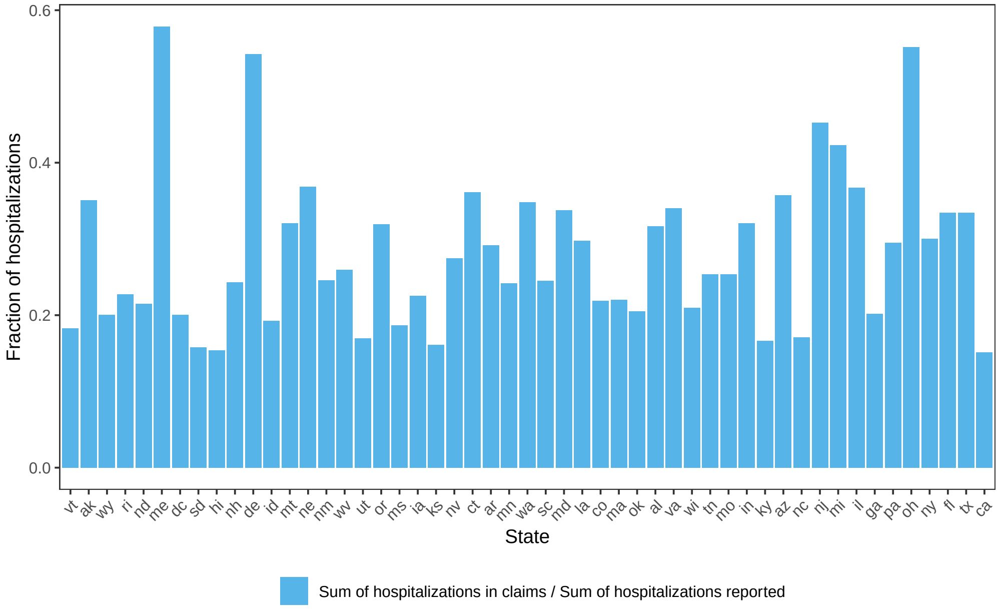

Fig S2. Fraction of reported hospitalizations captured by inpatient claims.

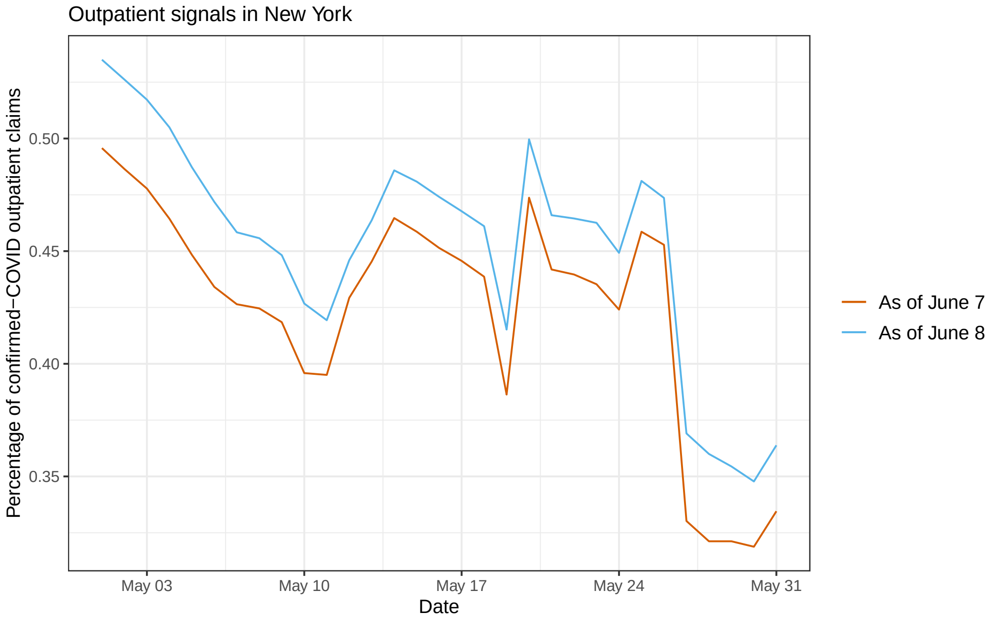

Fig S3. Two versions of the outpatient signal during May 2021, as of June 7 and June 8.

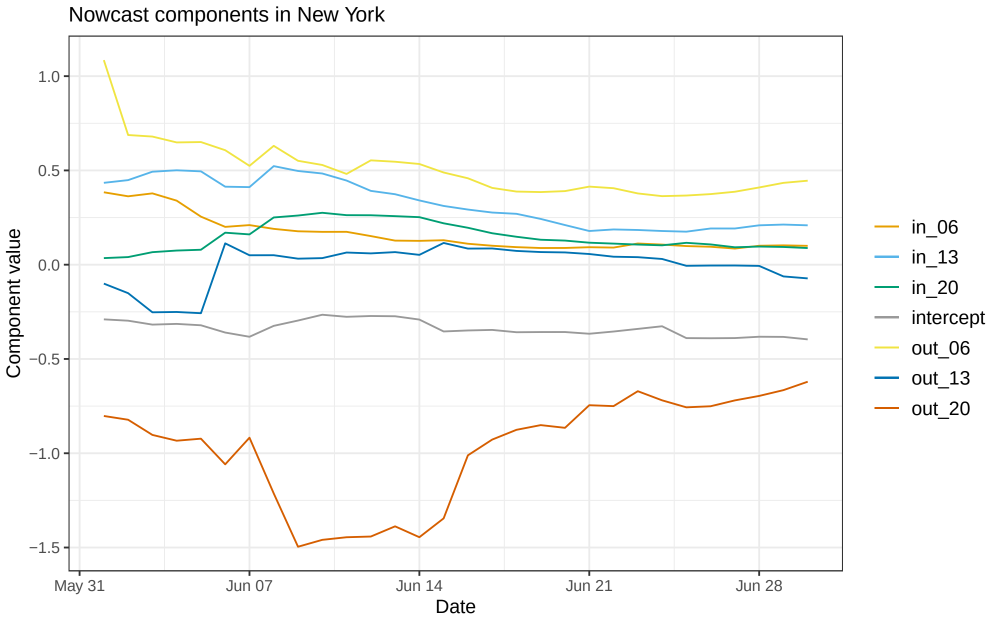

Fig S4. Contributions of each feature to the nowcasts during June 2021.

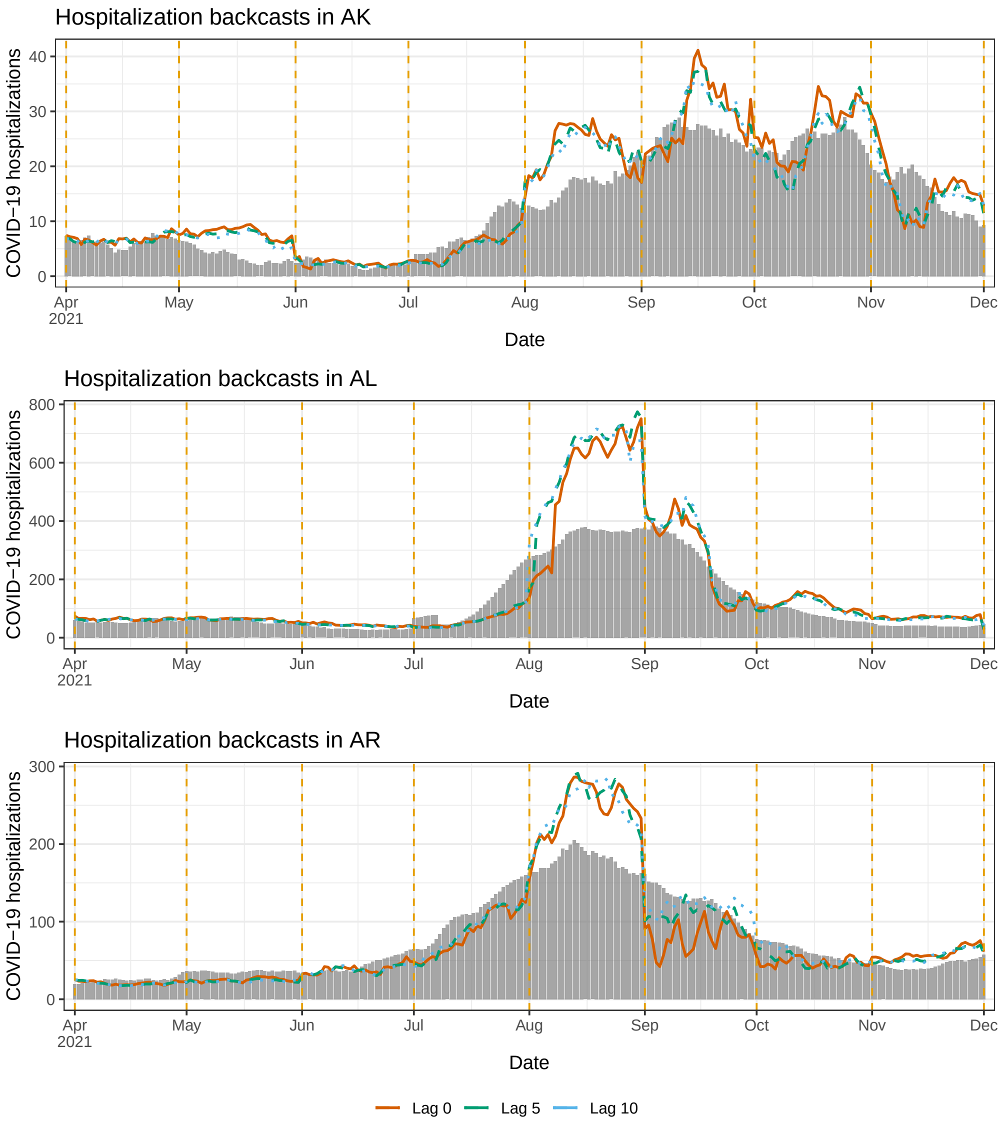

Fig S5. Backcasts from the mixed model in scenario 1, for AL, AK, AZ.

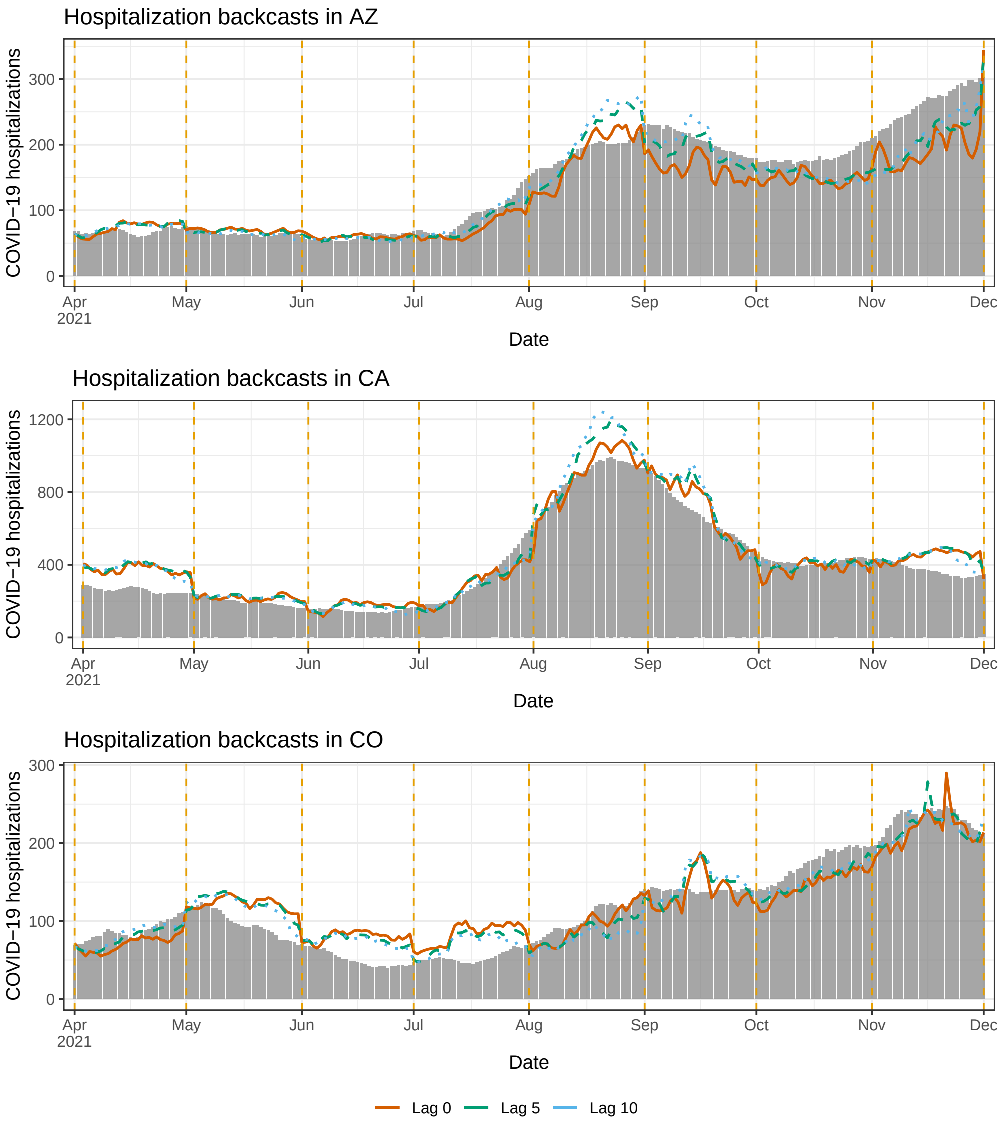

Fig S6. Backcasts from the mixed model in scenario 1, for AR, CA, CO.

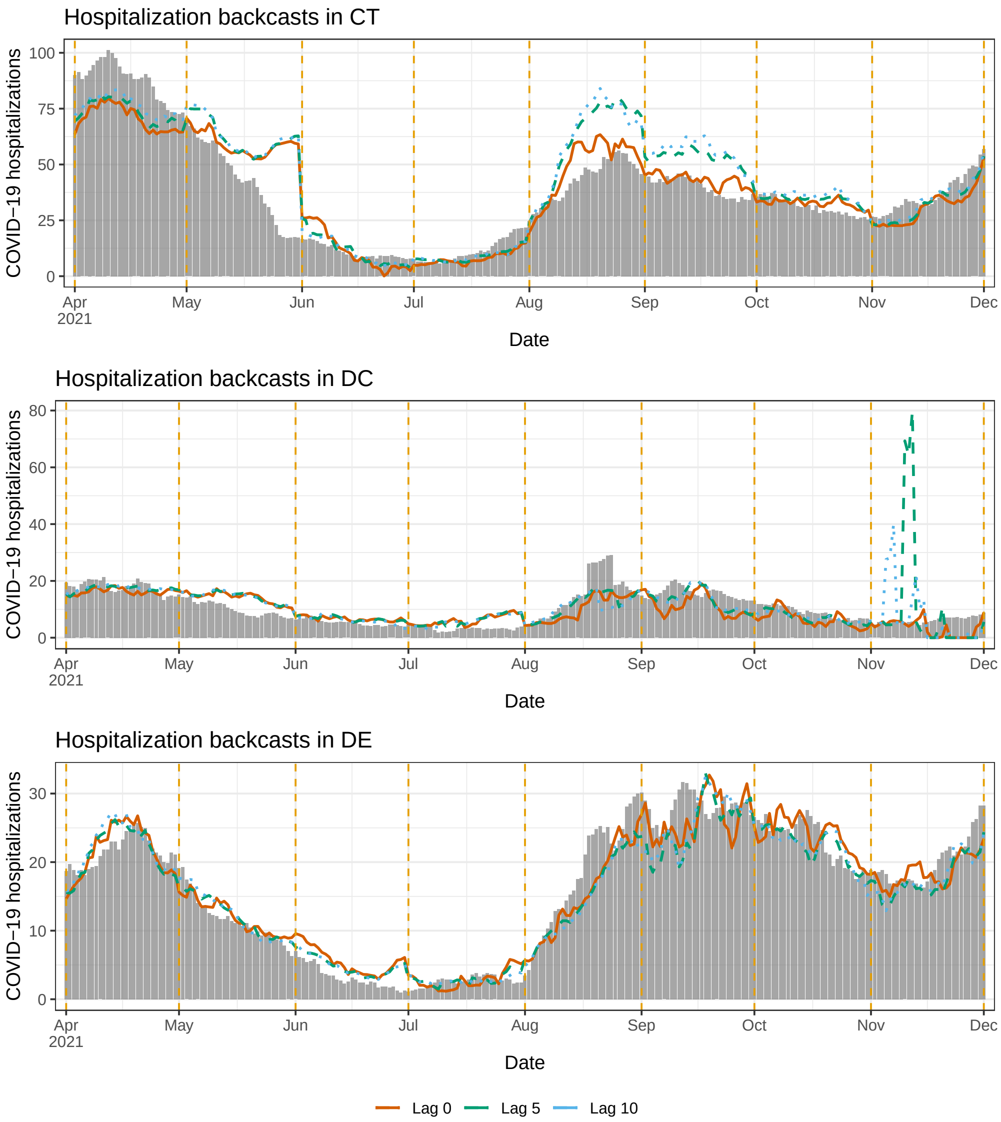

Fig S7. Backcasts from the mixed model in scenario 1, for CT, DC, DE.

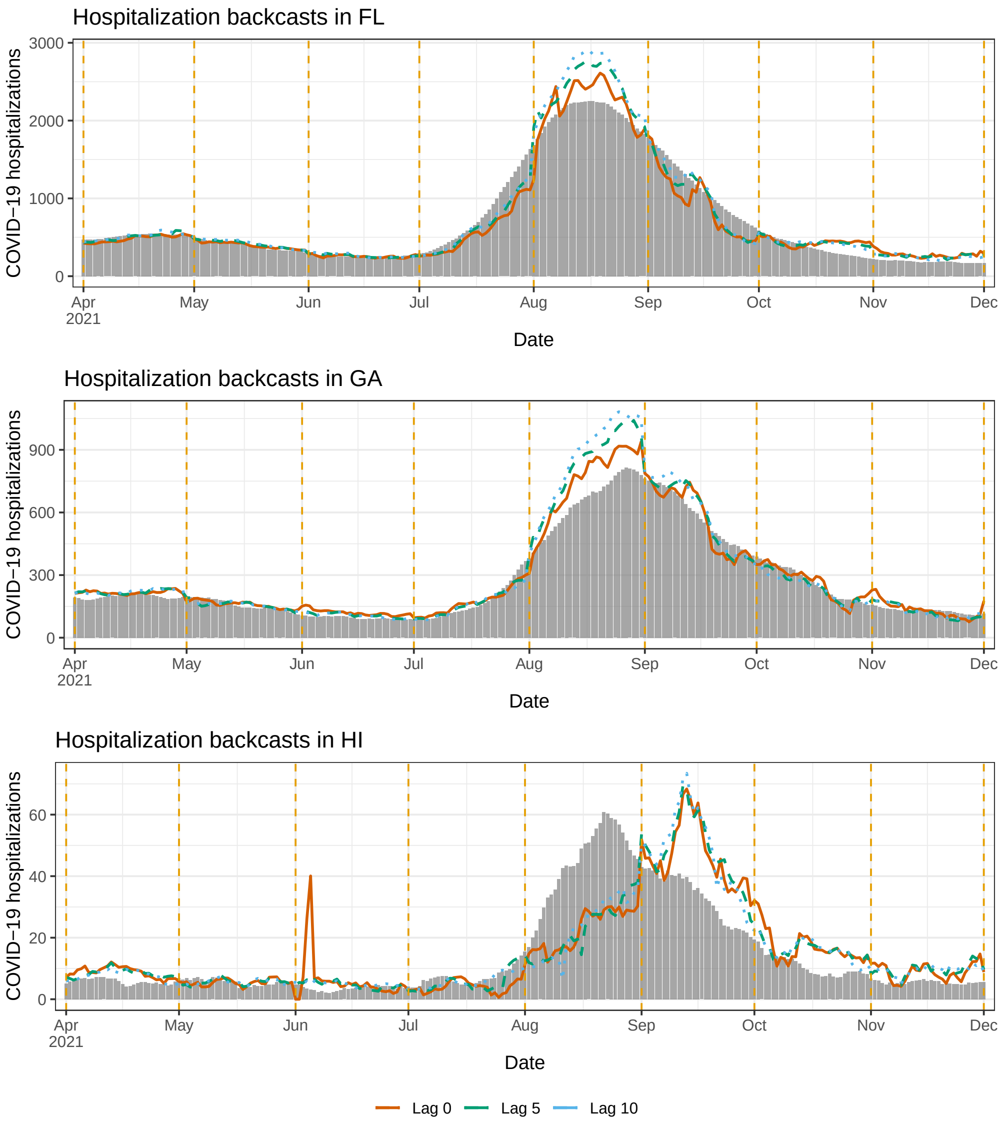

Fig S8. Backcasts from the mixed model in scenario 1, for FL, GA, HI.

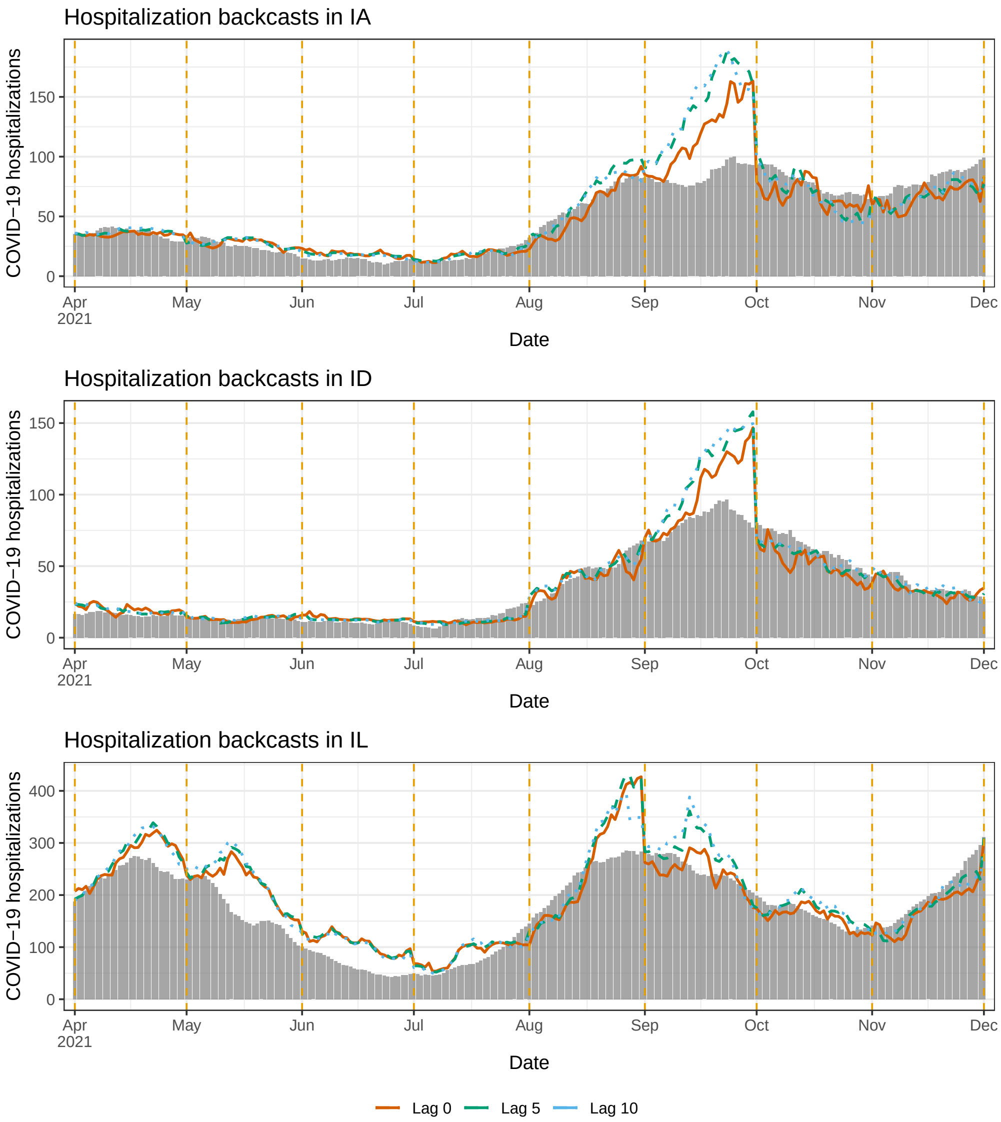

Fig S9. Backcasts from the mixed model in scenario 1, for IA, ID, IL.

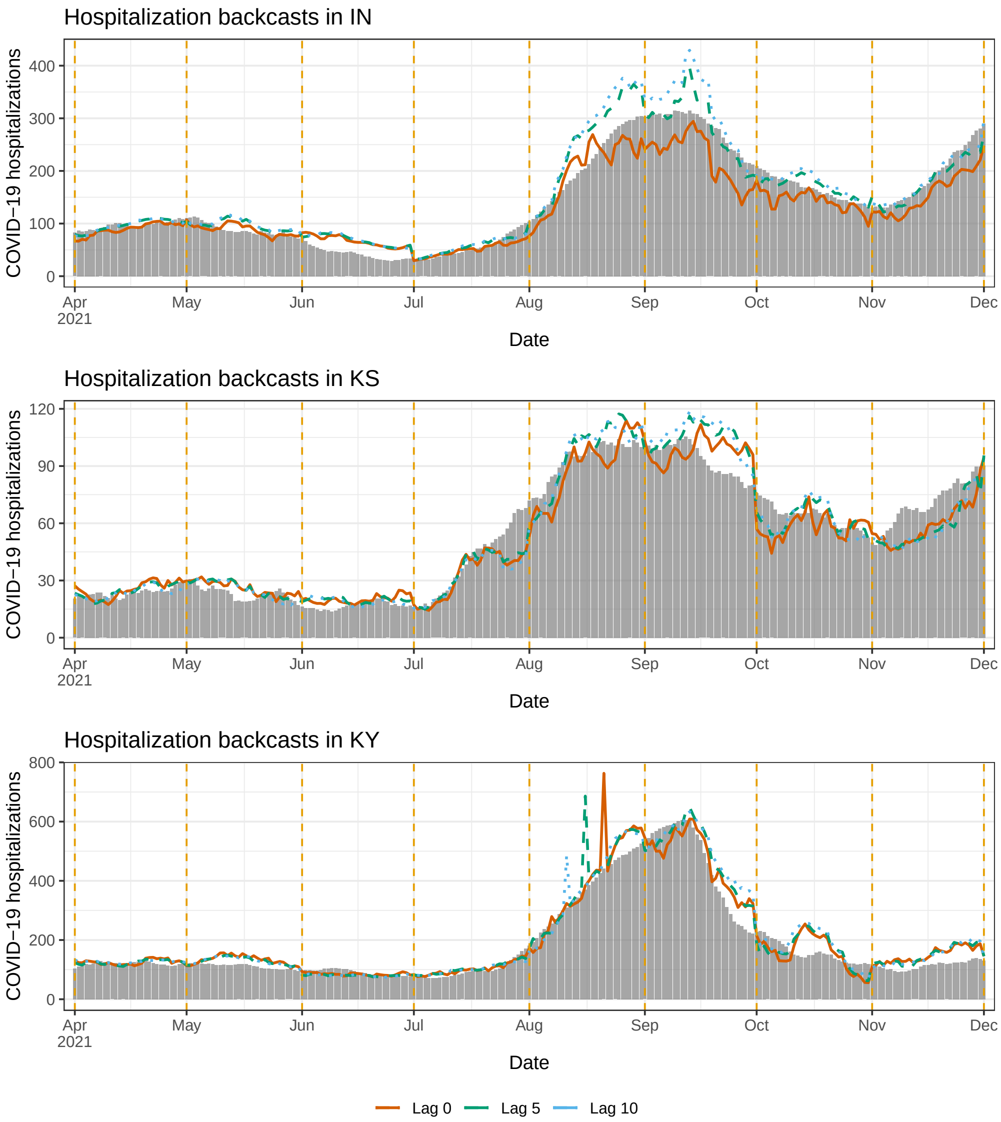

Fig S10. Backcasts from the mixed model in scenario 1, for IN, KS, KY.

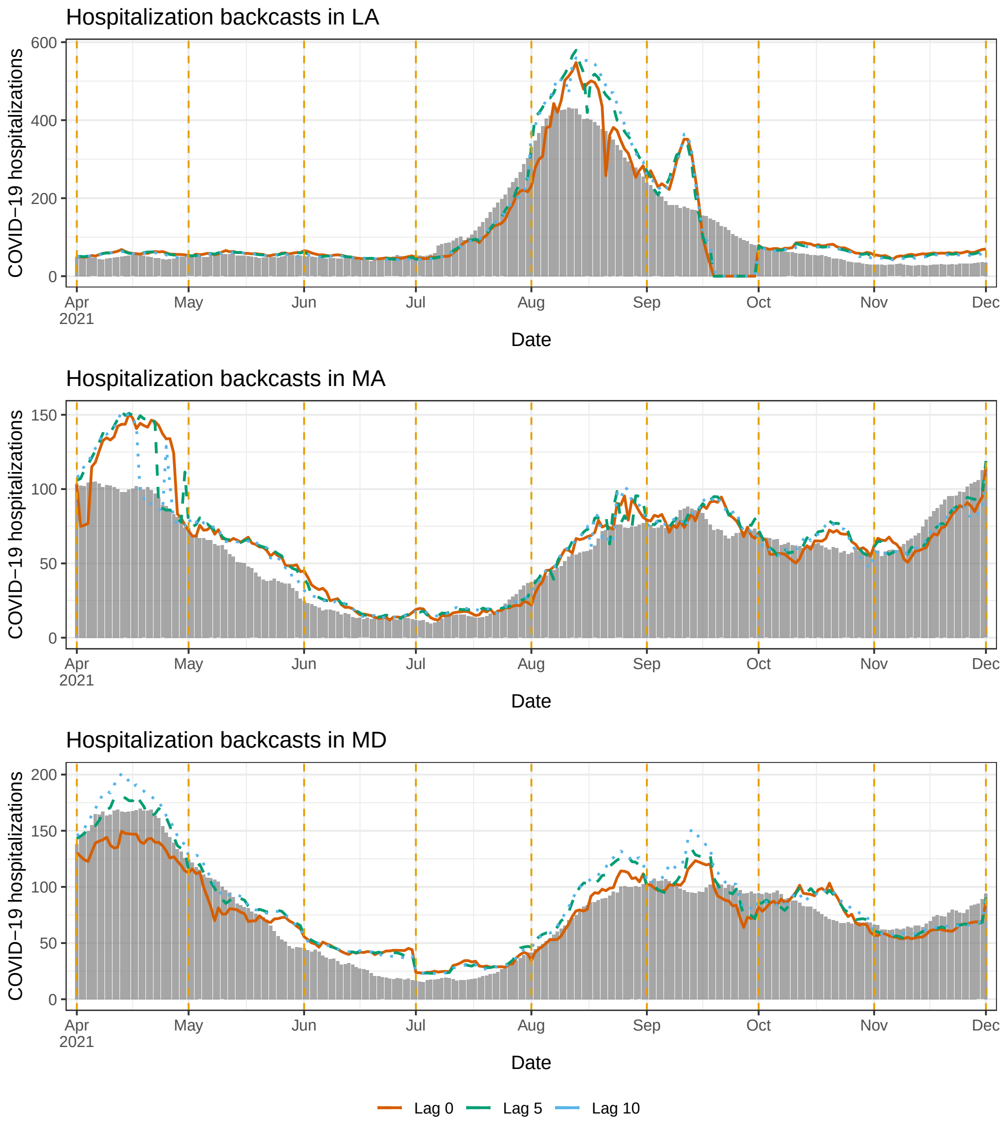

Fig S11. Backcasts from the mixed model in scenario 1, for LA, MA, MD.

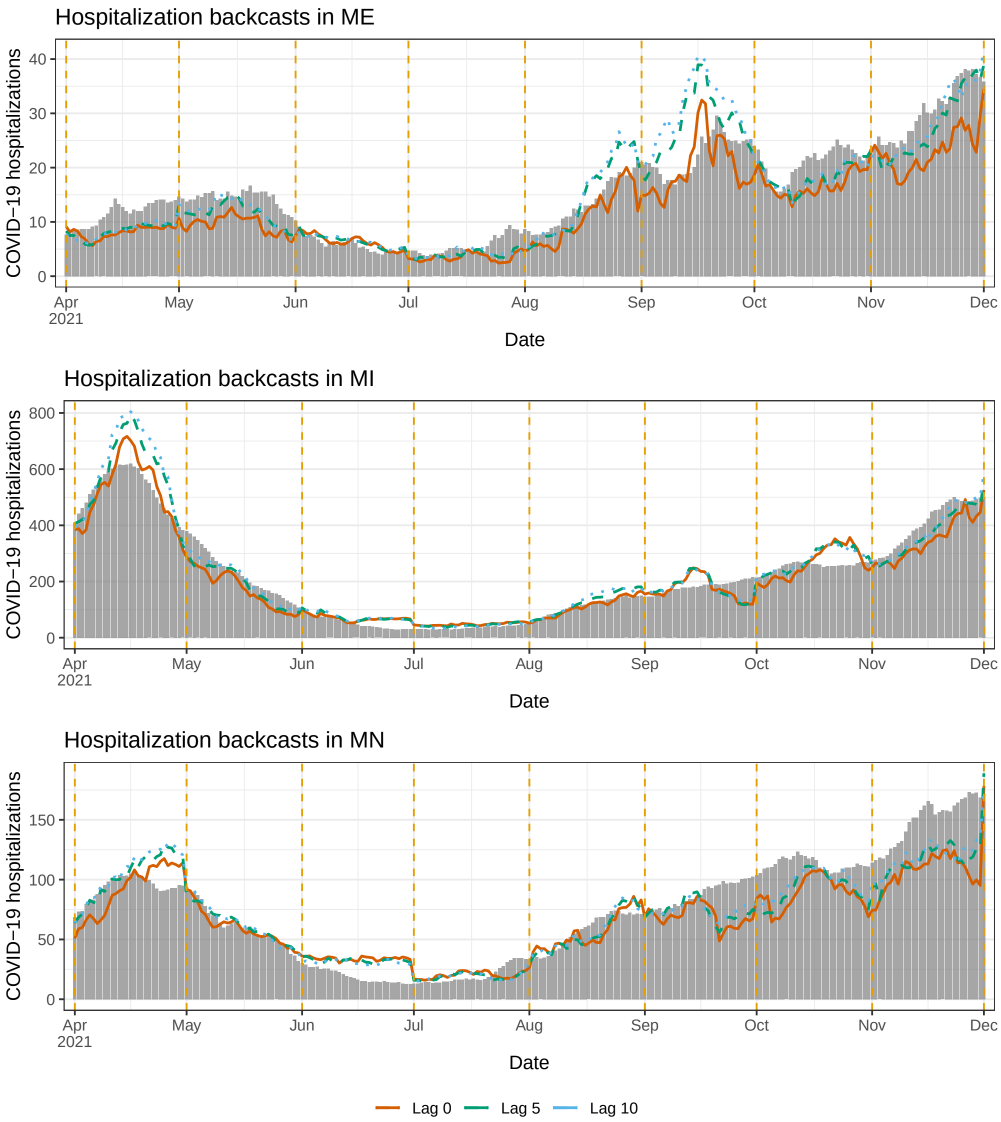

Fig S12. Backcasts from the mixed model in scenario 1, for ME, MI, MN.

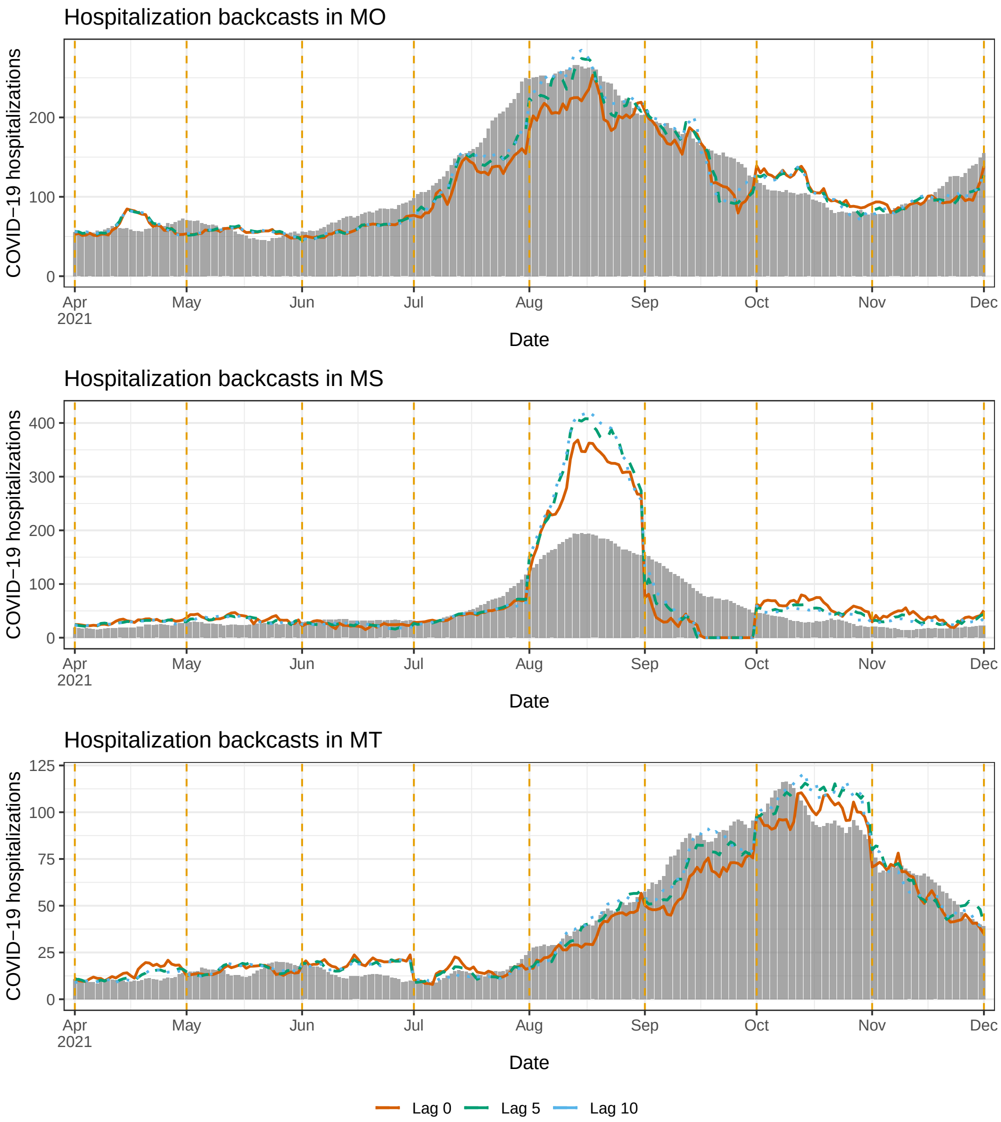

Fig S13. Backcasts from the mixed model in scenario 1, for MO, MS, MT.

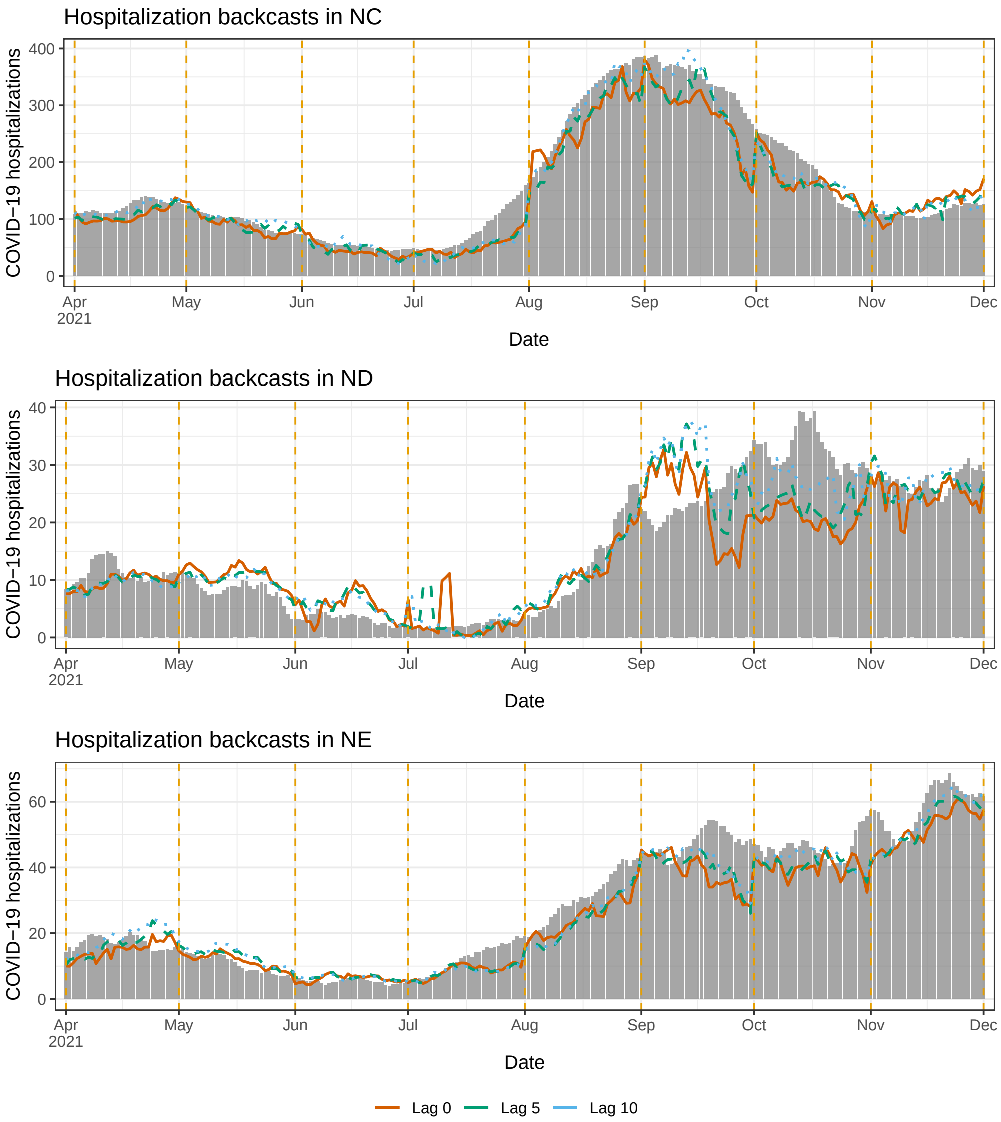

Fig S14. Backcasts from the mixed model in scenario 1, for NC, ND, NE.

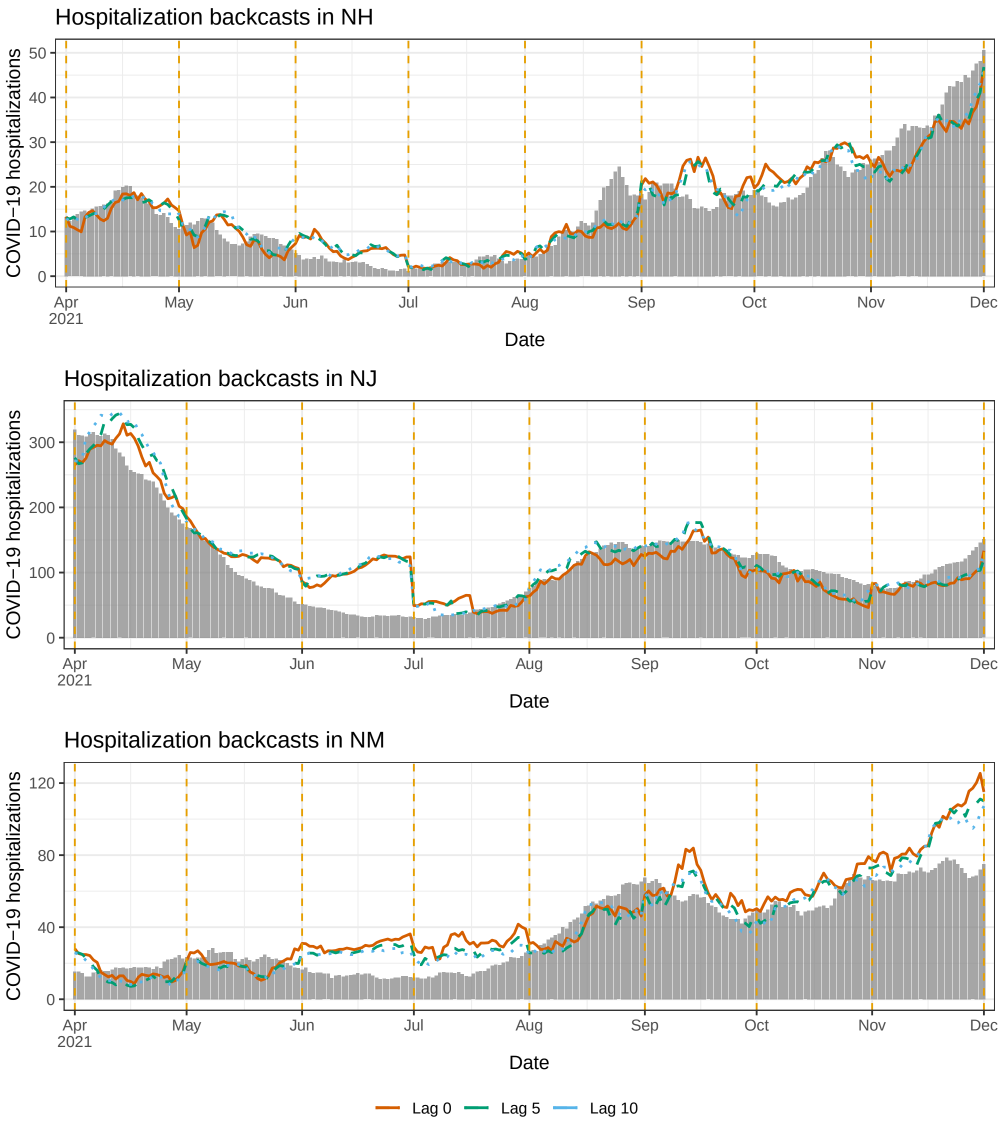

Fig S15. Backcasts from the mixed model in scenario 1, for NH, NJ, NM.

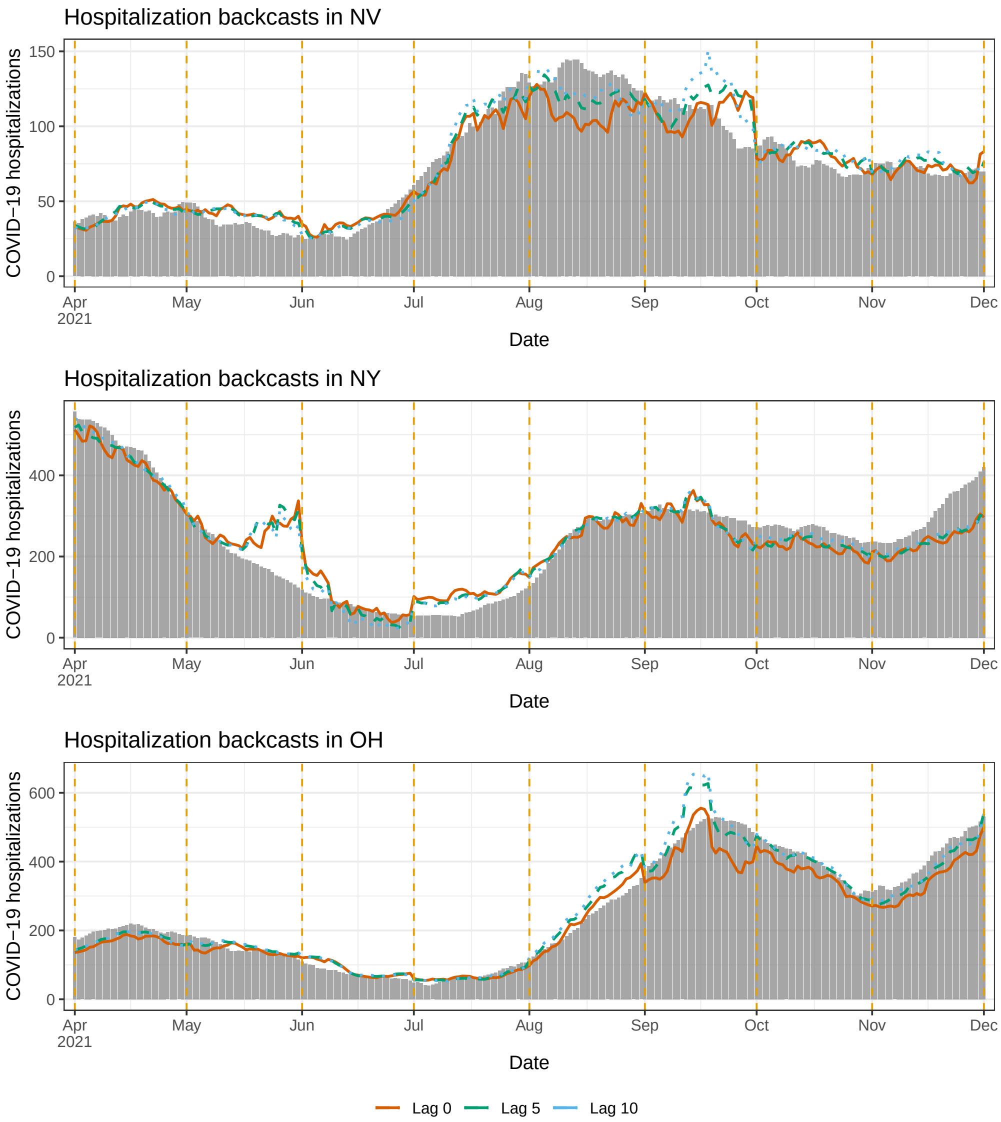

Fig S16. Backcasts from the mixed model in scenario 1, for NV, NY, OH.

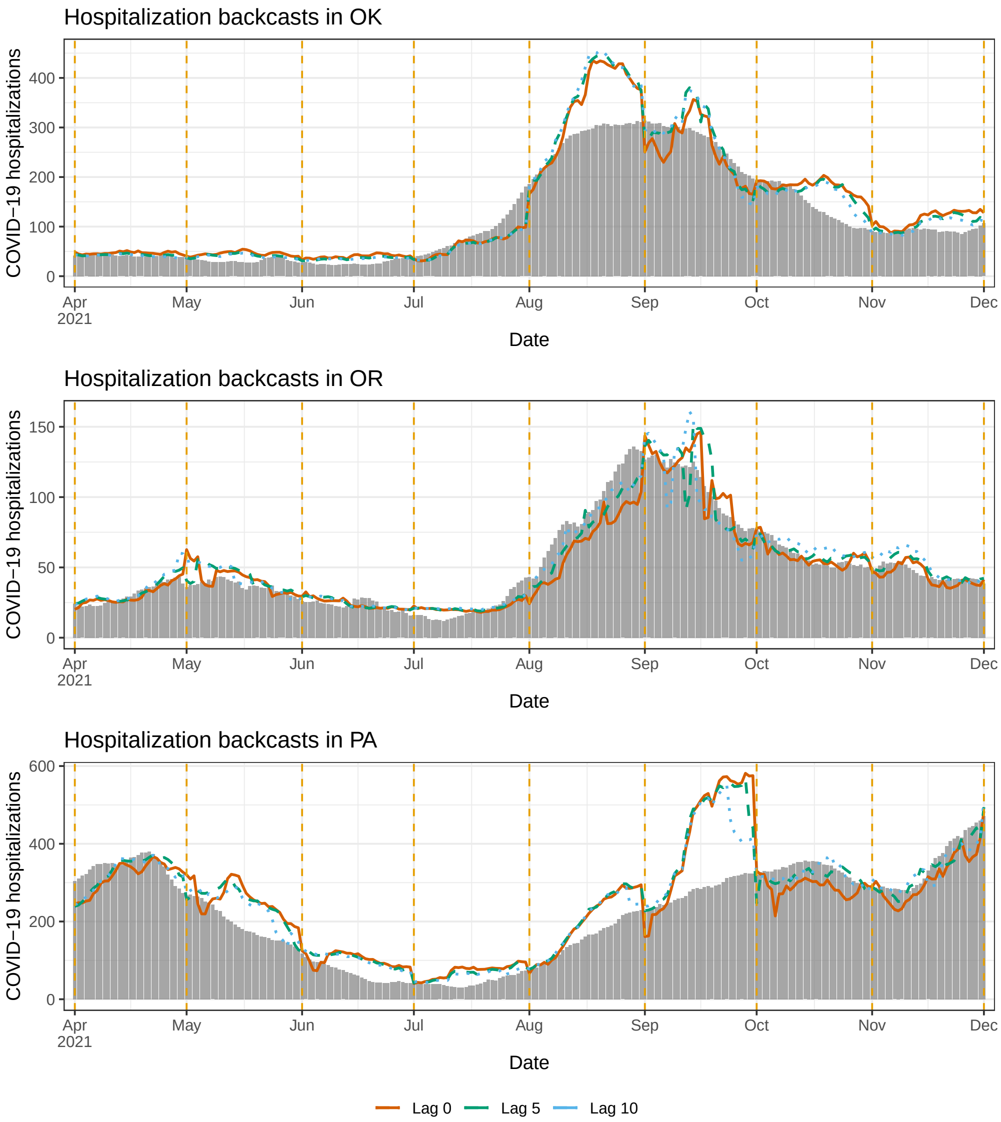

Fig S17. Backcasts from the mixed model in scenario 1, for OK, OR, PA.

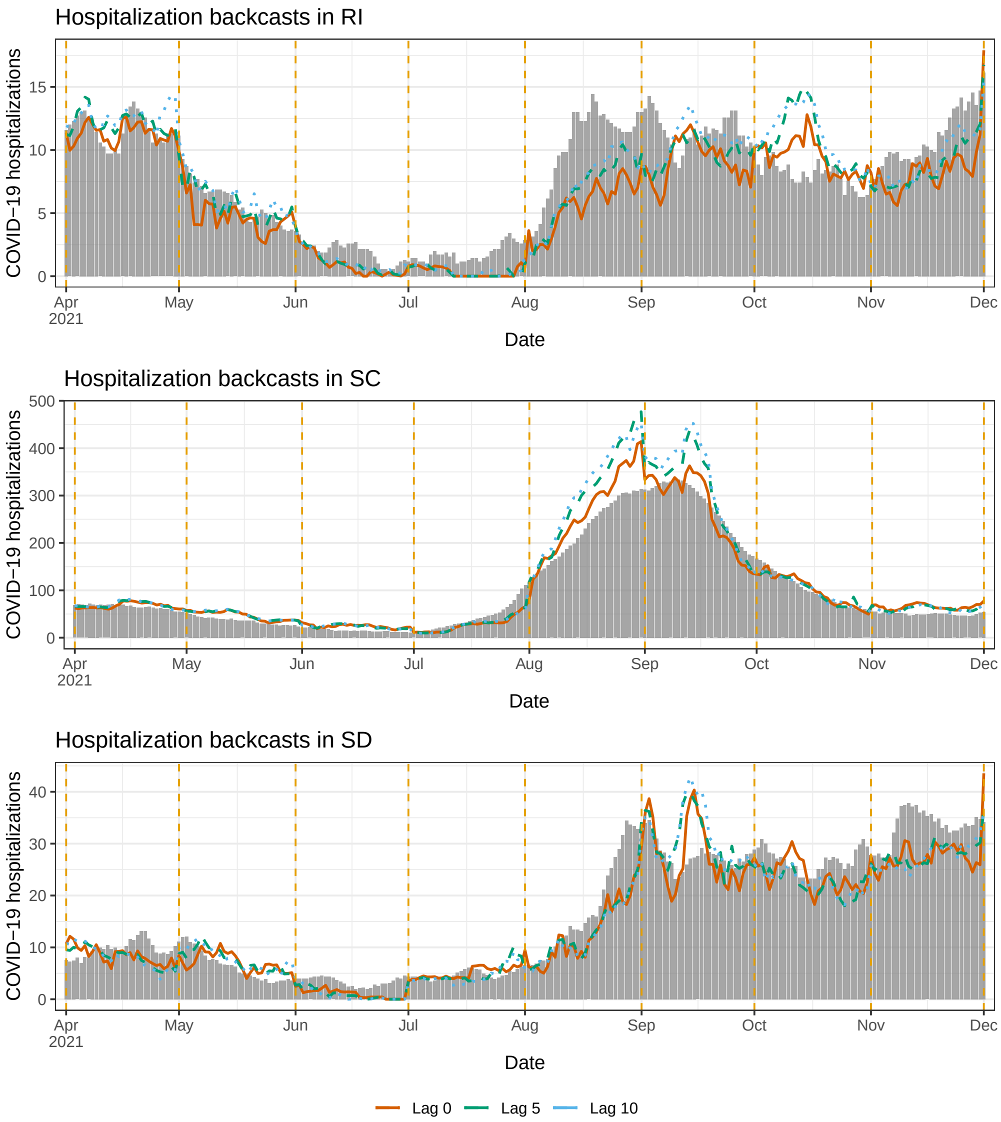

Fig S18. Backcasts from the mixed model in scenario 1, for RI, SC, SD.

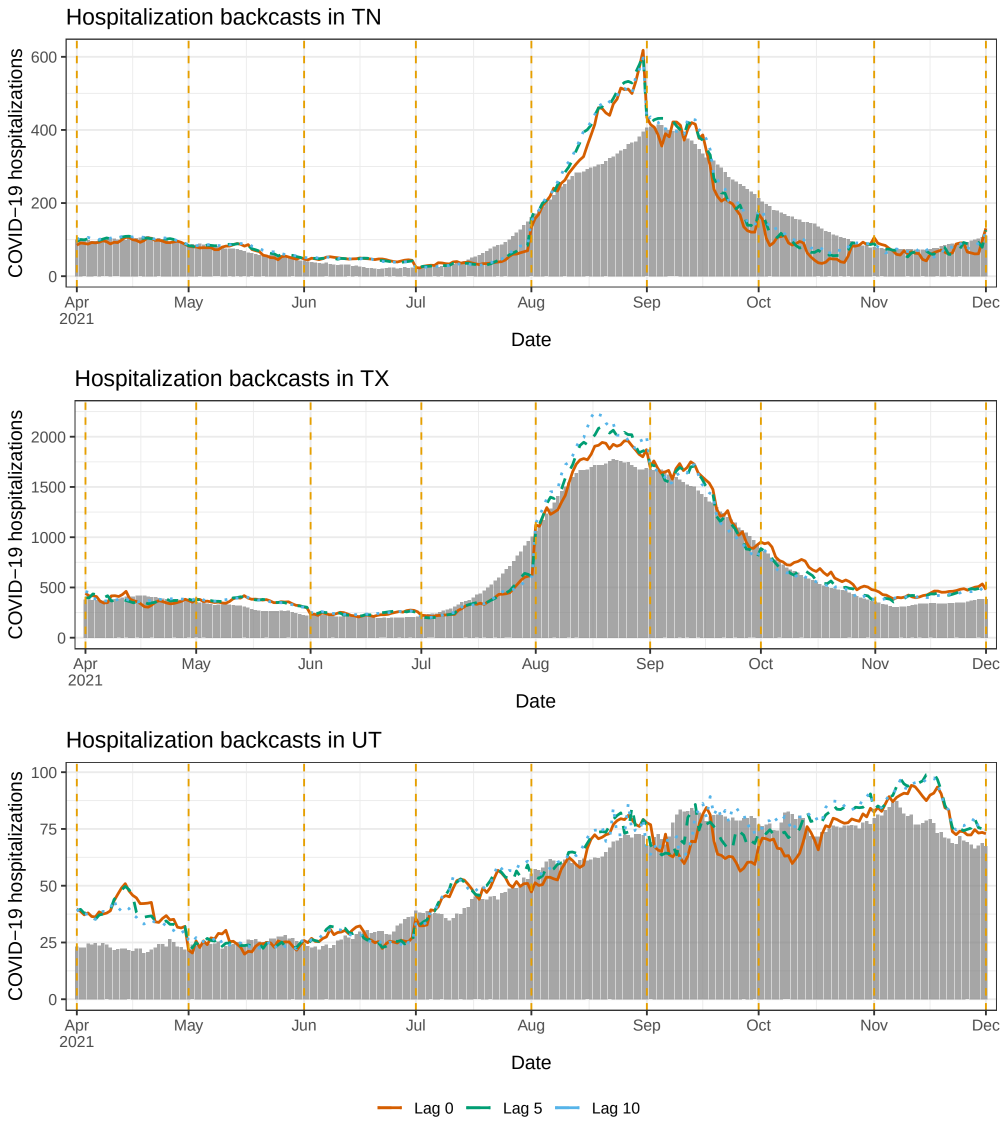

Fig S19. Backcasts from the mixed model in scenario 1, for TN, TX, UT.

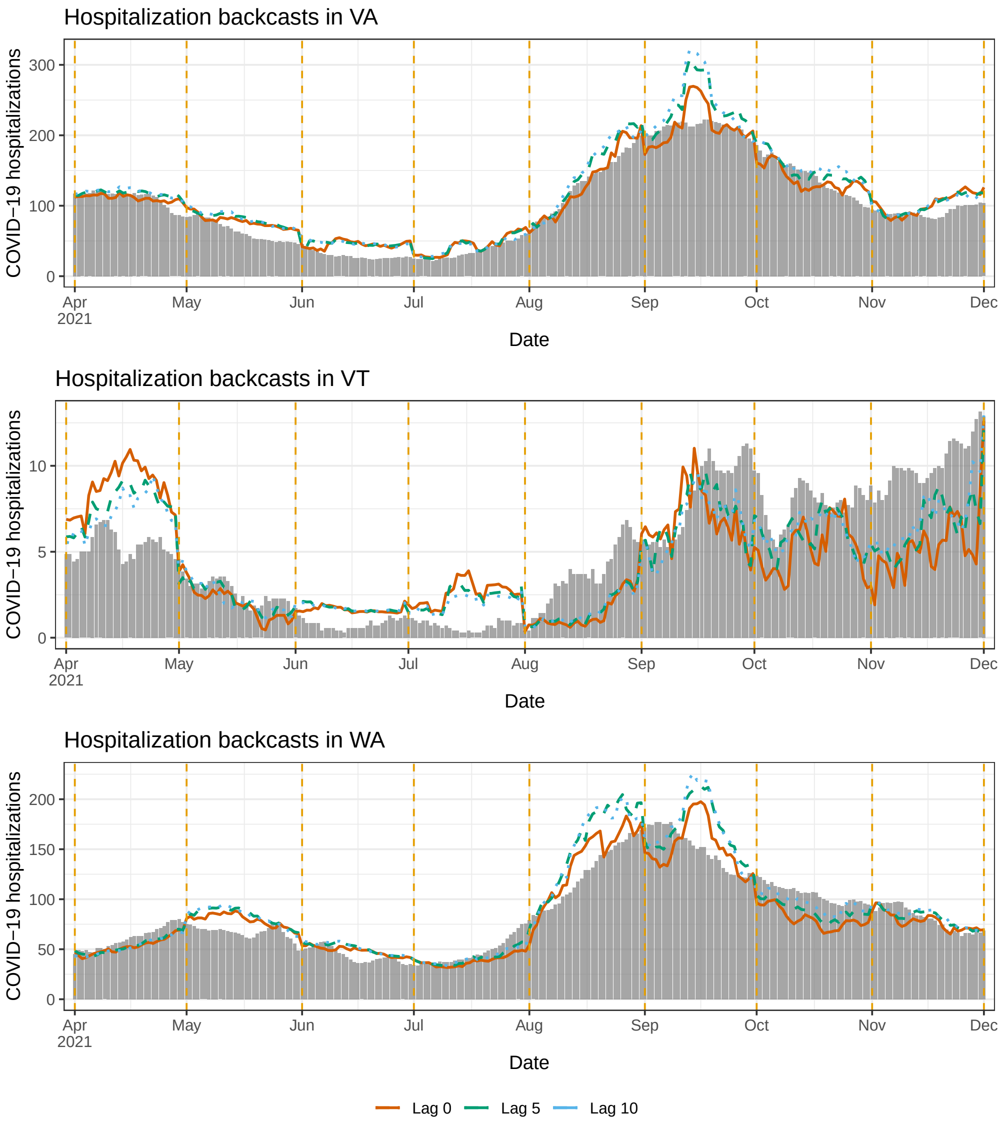

Fig S20. Backcasts from the mixed model in scenario 1, for VA, VT, WA.

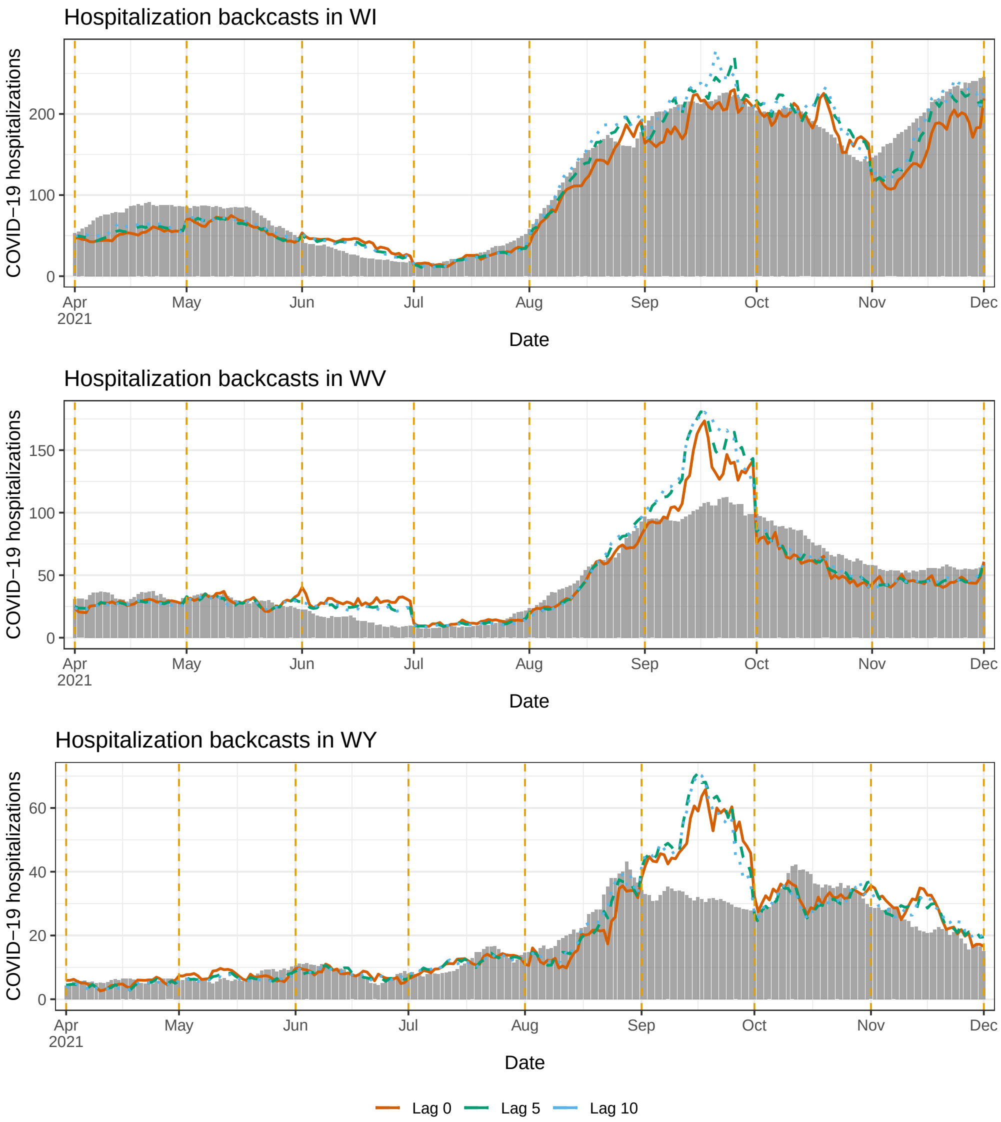

Fig S21. Backcasts from the mixed model in scenario 1, for WI, WV, WY.

D Scenario 2: full set of backcasts

Figs S22–S38 display backcasts at lag 0 (i.e., nowcasts), 5, and 10, for all 50 US states and DC, made by the mixed model in scenario 2, the no-update period. The format follows Fig 7 in the main paper.

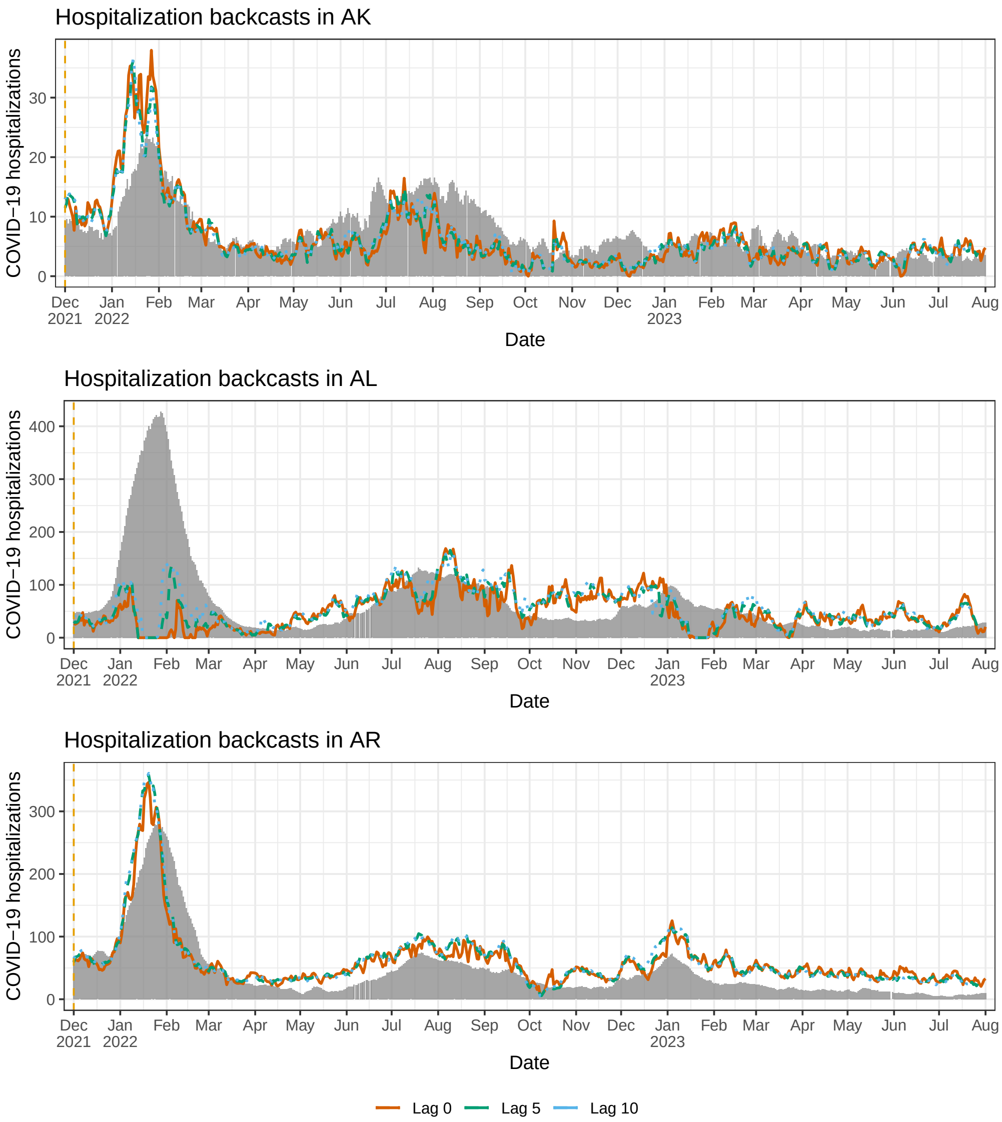

Fig S22. Backcasts from the mixed model in scenario 2, for AL, AK, AZ.

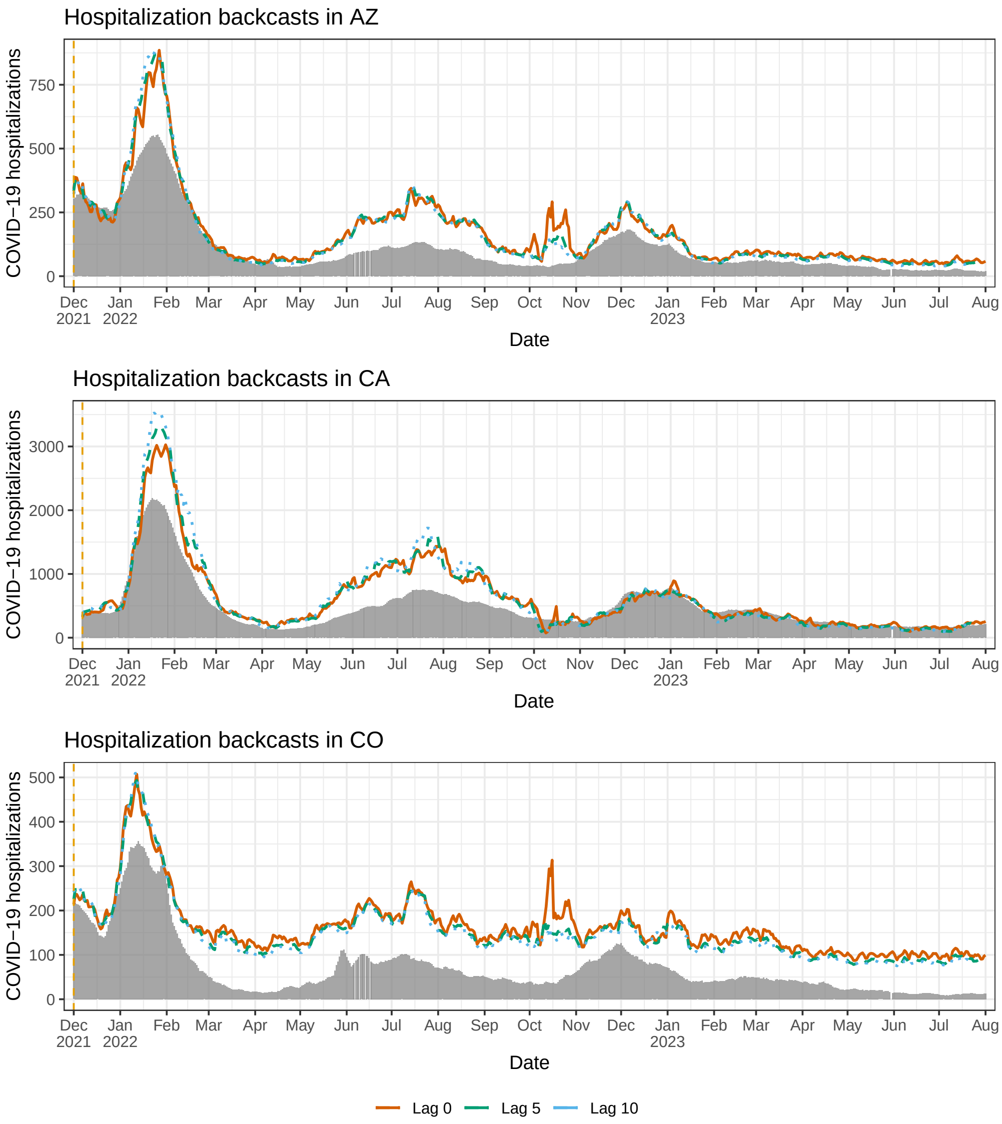

Fig S23. Backcasts from the mixed model in scenario 2, for AR, CA, CO.

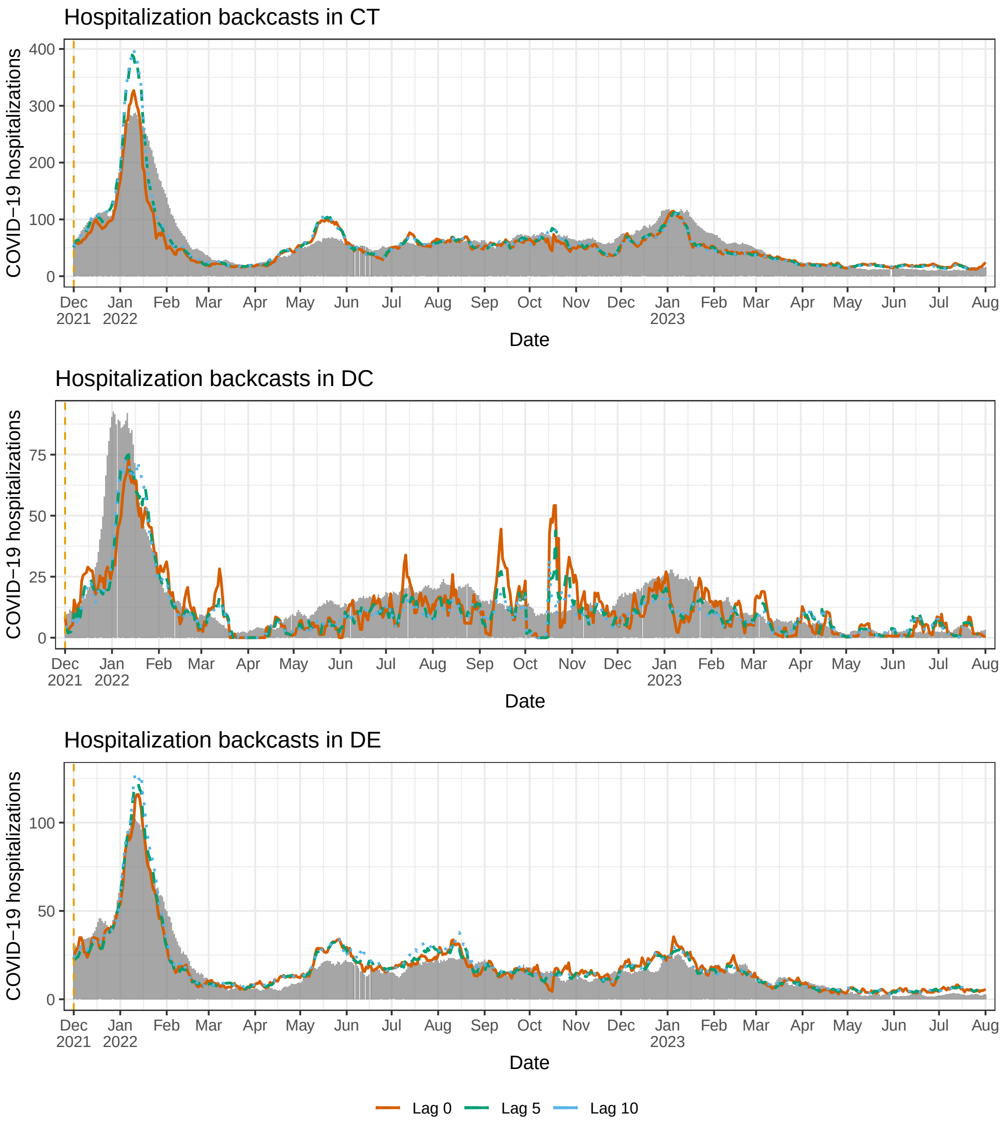

Fig S24. Backcasts from the mixed model in scenario 2, for CT, DC, DE.

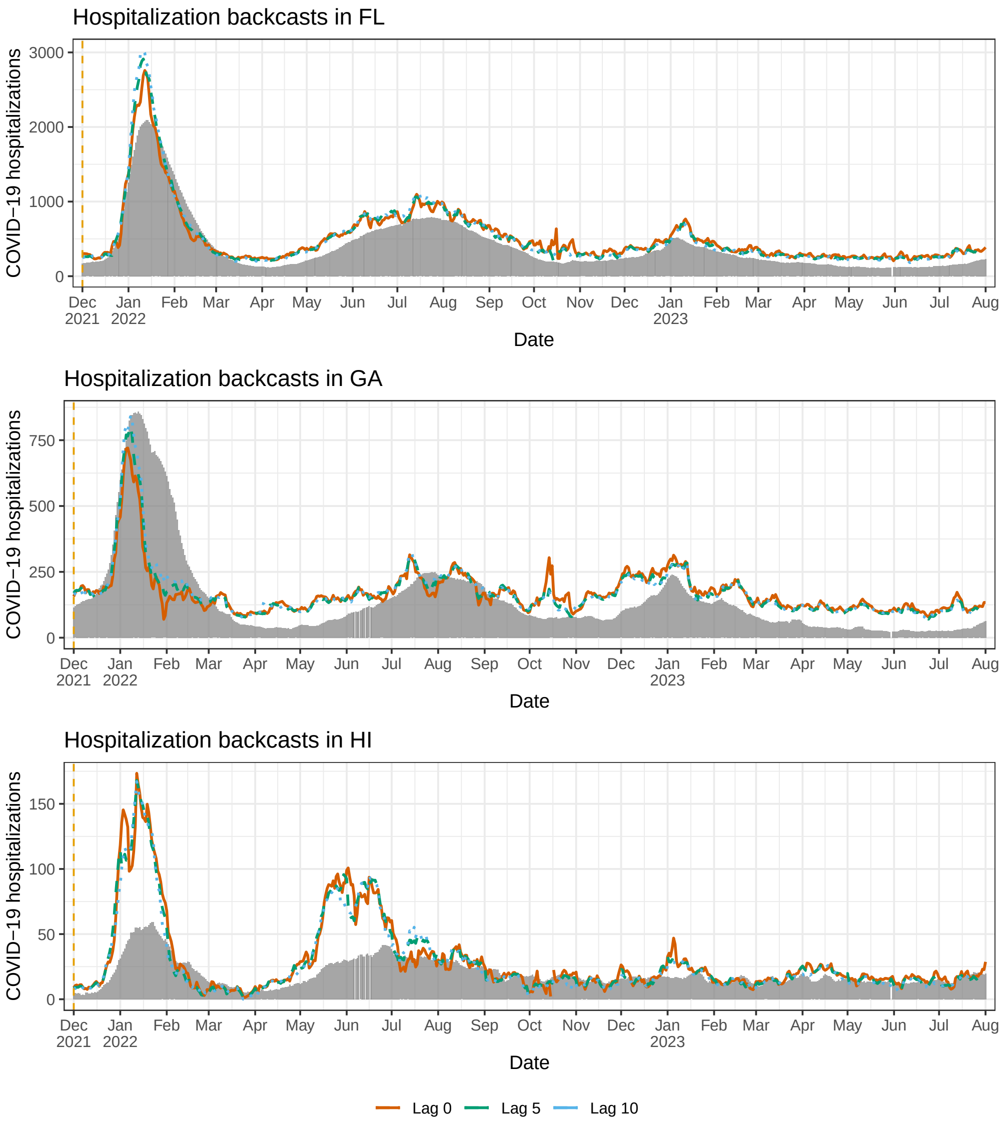

Fig S25. Backcasts from the mixed model in scenario 2, for FL, GA, HI.

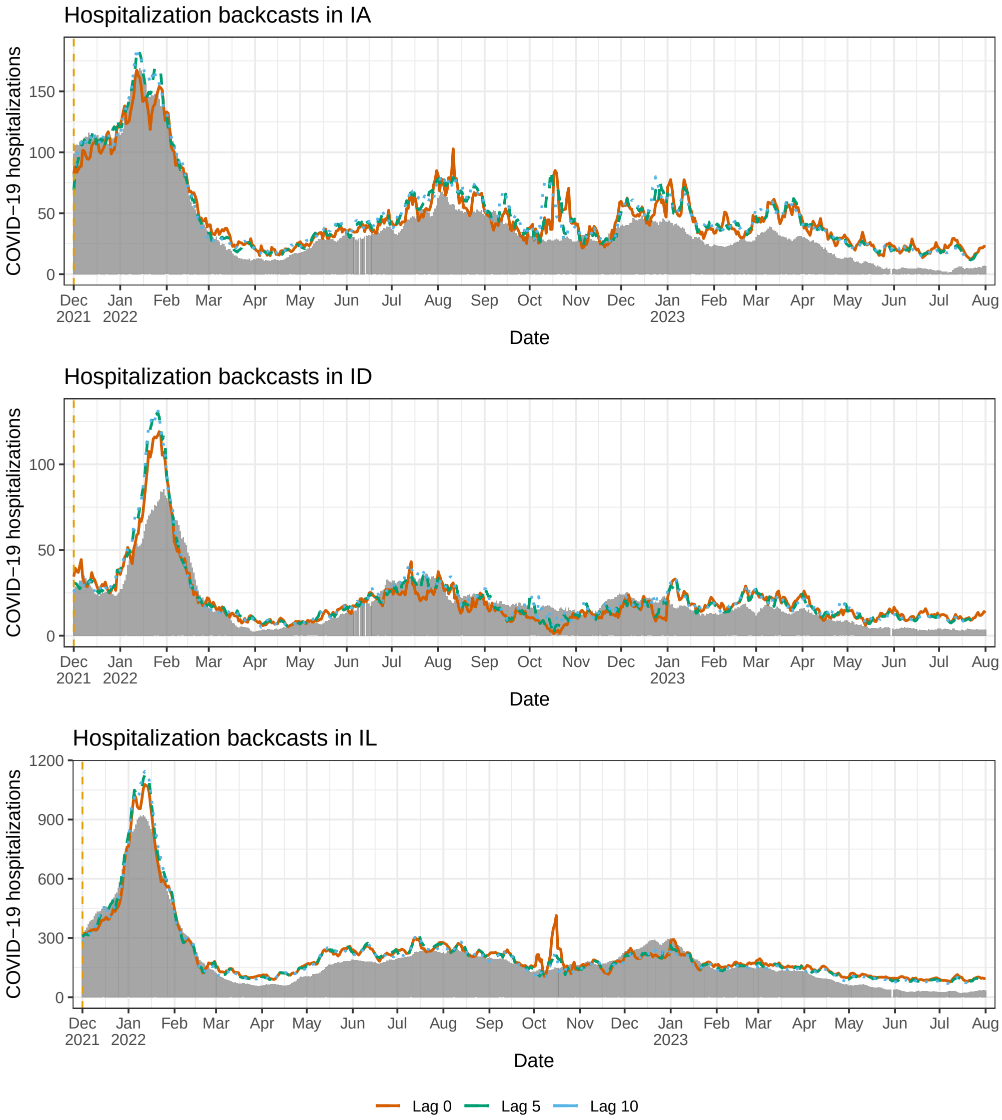

Fig S26. Backcasts from the mixed model in scenario 2, for IA, ID, IL.

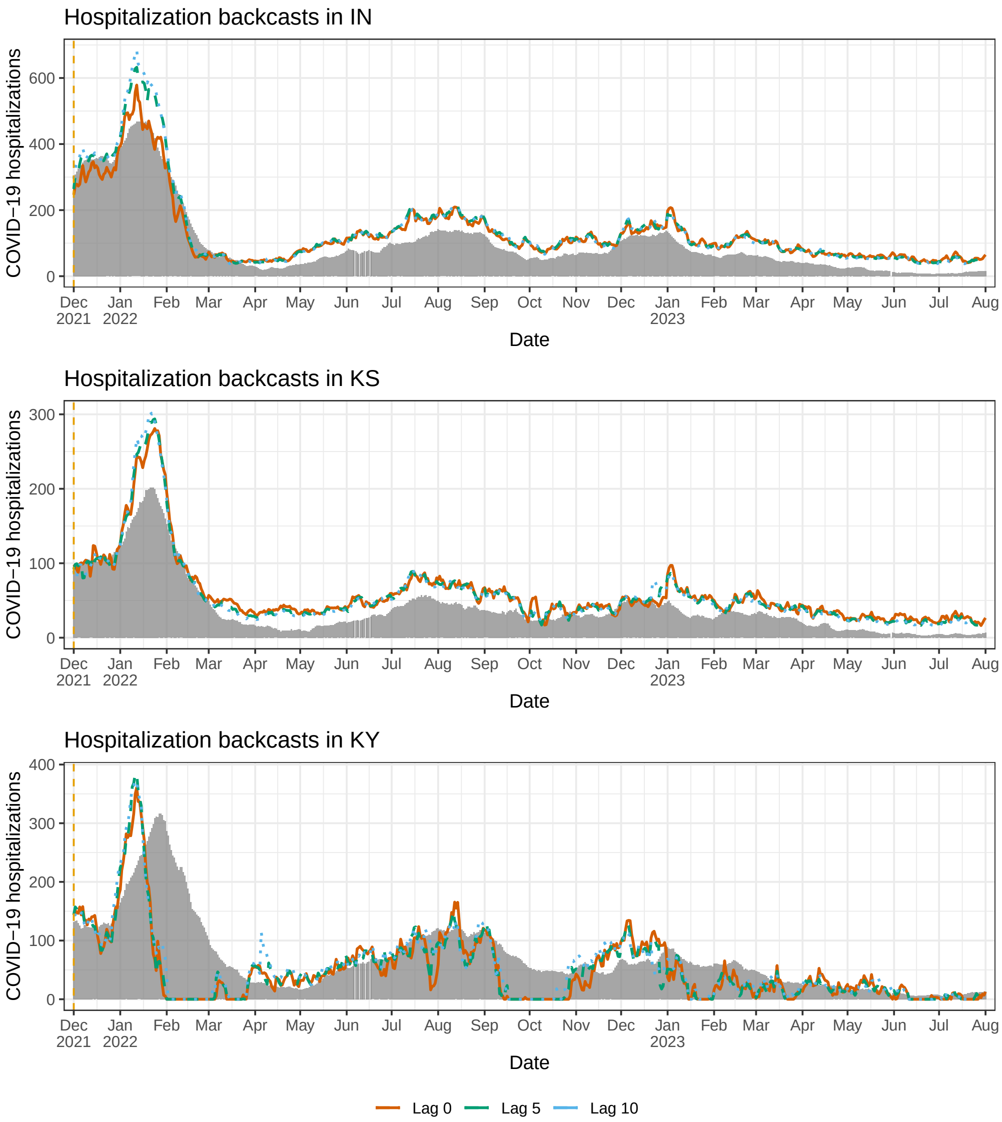

Fig S27. Backcasts from the mixed model in scenario 2, for IN, KS, KY.

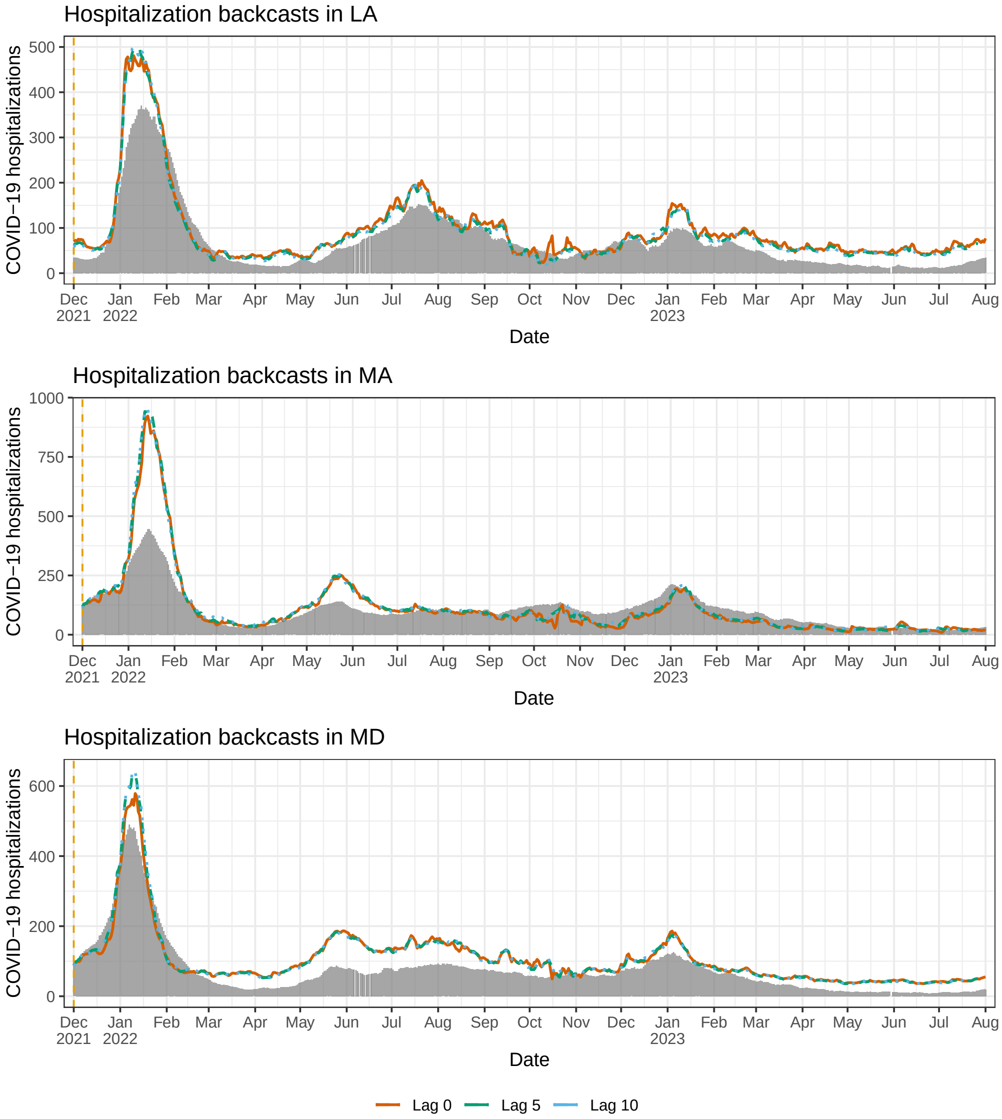

Fig S28. Backcasts from the mixed model in scenario 2, for LA, MA, MD.

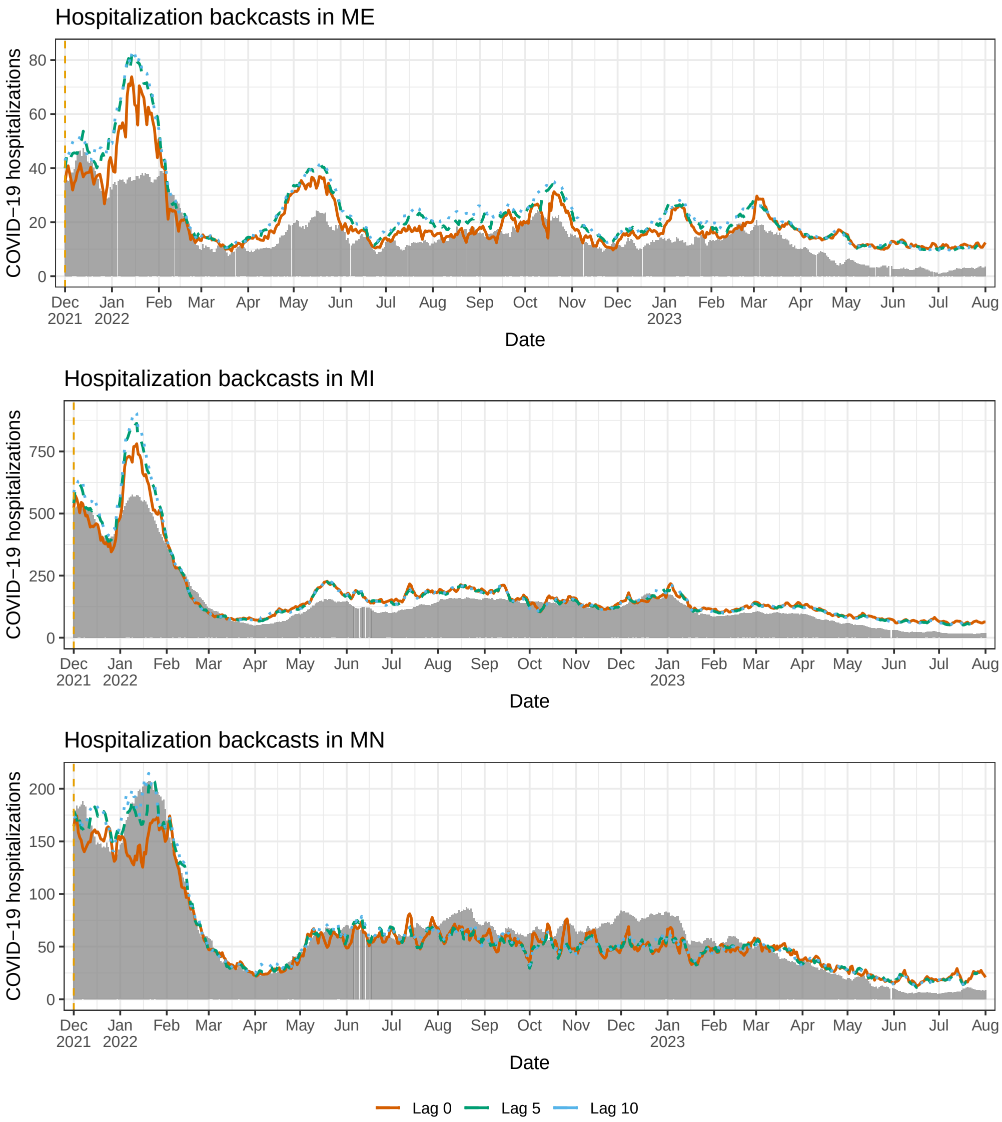

Fig S29. Backcasts from the mixed model in scenario 2, for ME, MI, MN.

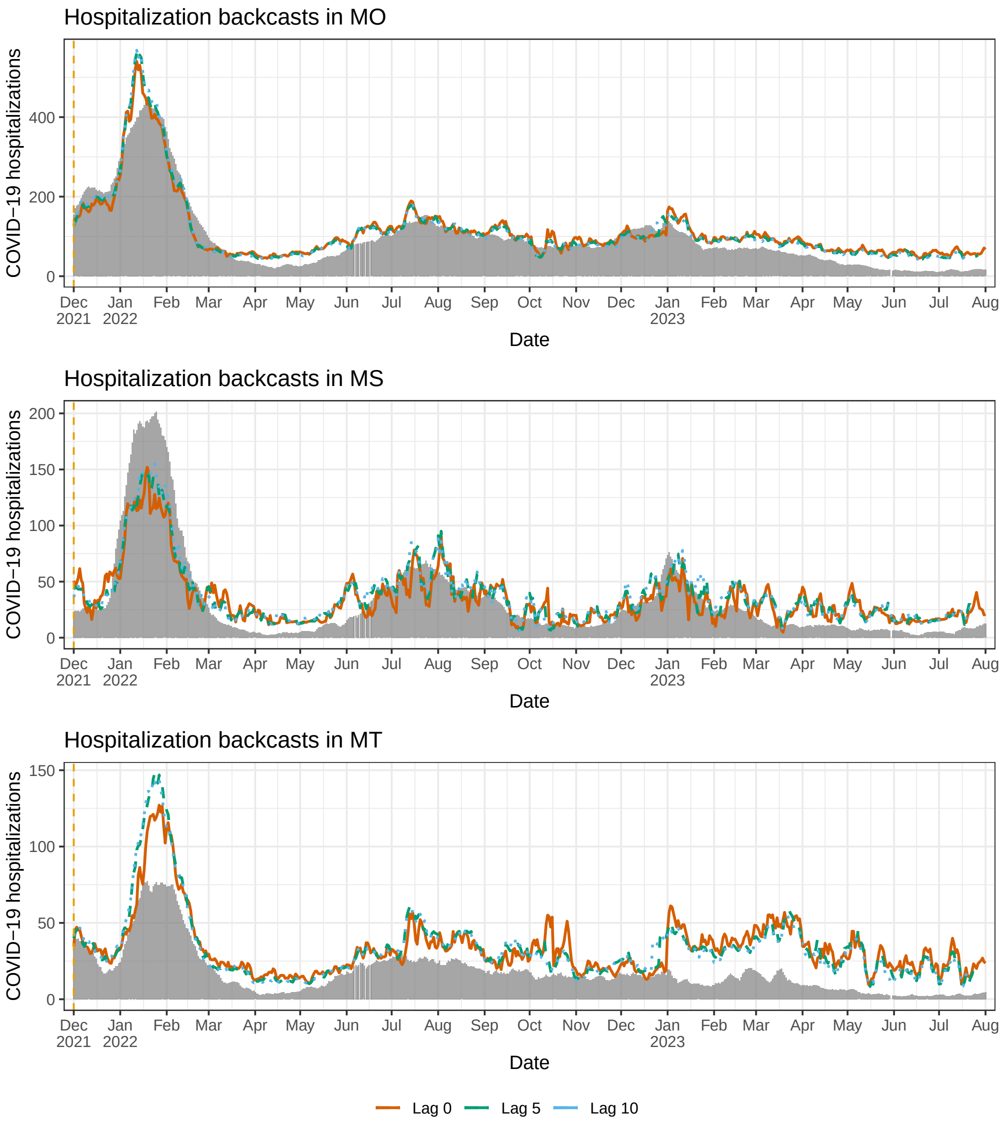

Fig S30. Backcasts from the mixed model in scenario 2, for MO, MS, MT.

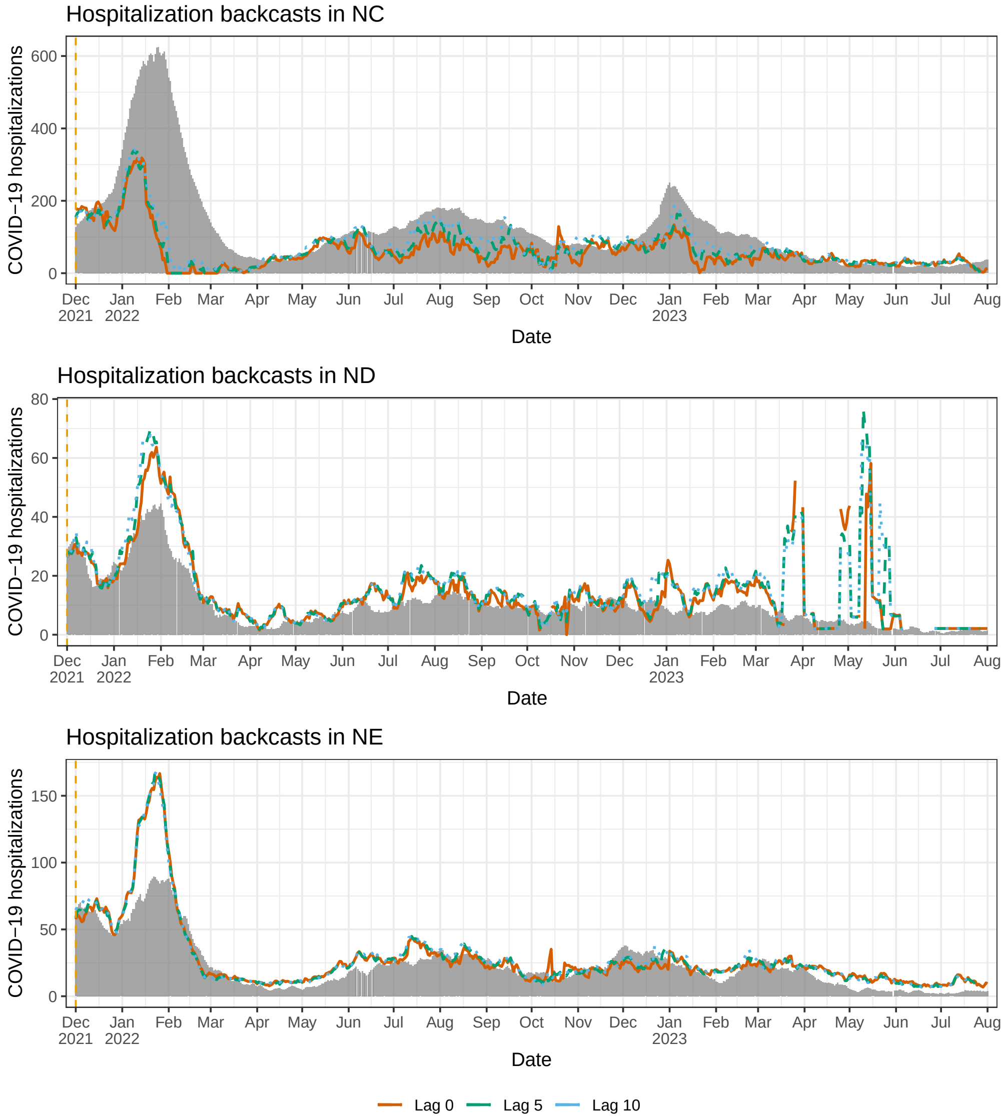

Fig S31. Backcasts from the mixed model in scenario 2, for NC, ND, NE.

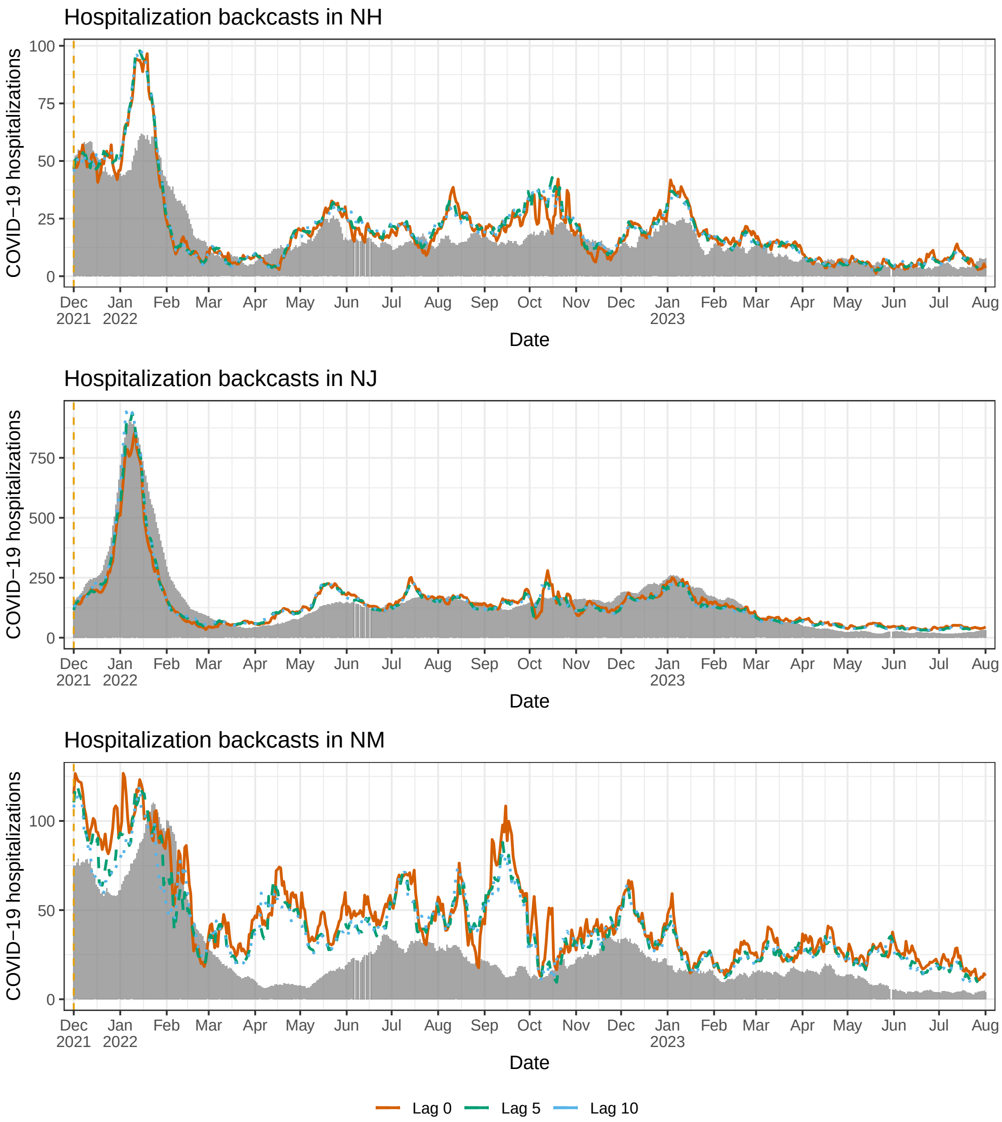

Fig S32. Backcasts from the mixed model in scenario 2, for NH, NJ, NM.

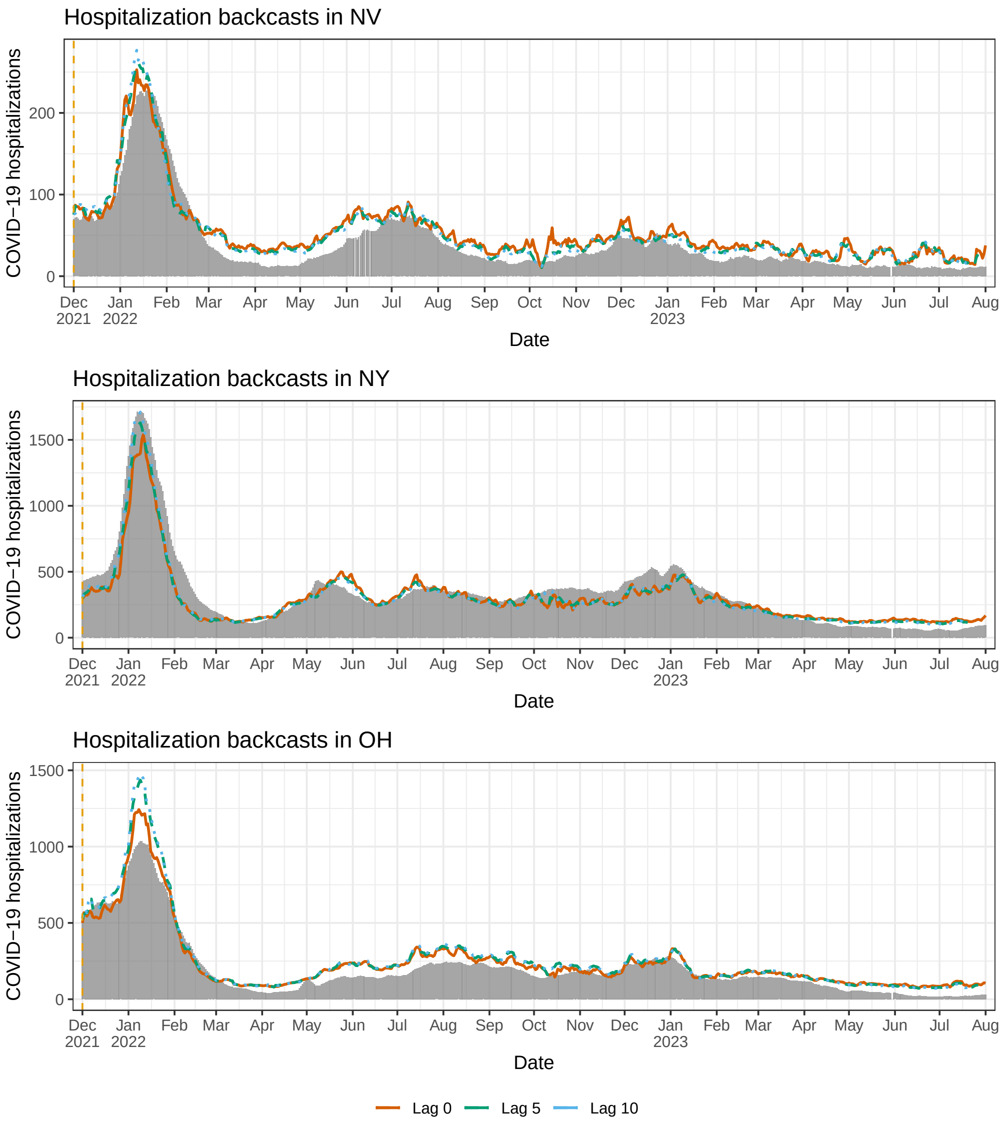

Fig S33. Backcasts from the mixed model in scenario 2, for NV, NY, OH.

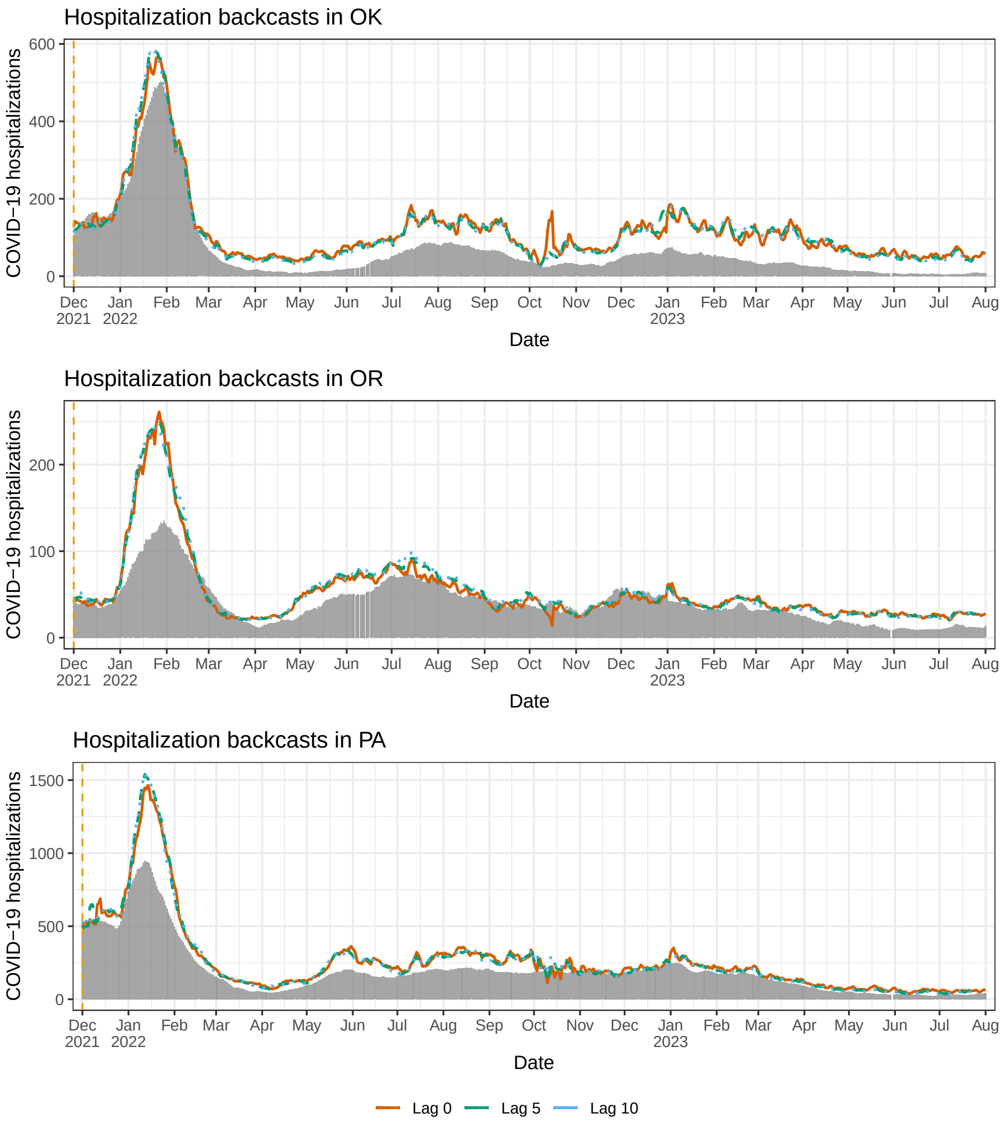

Fig S34. Backcasts from the mixed model in scenario 2, for OK, OR, PA.

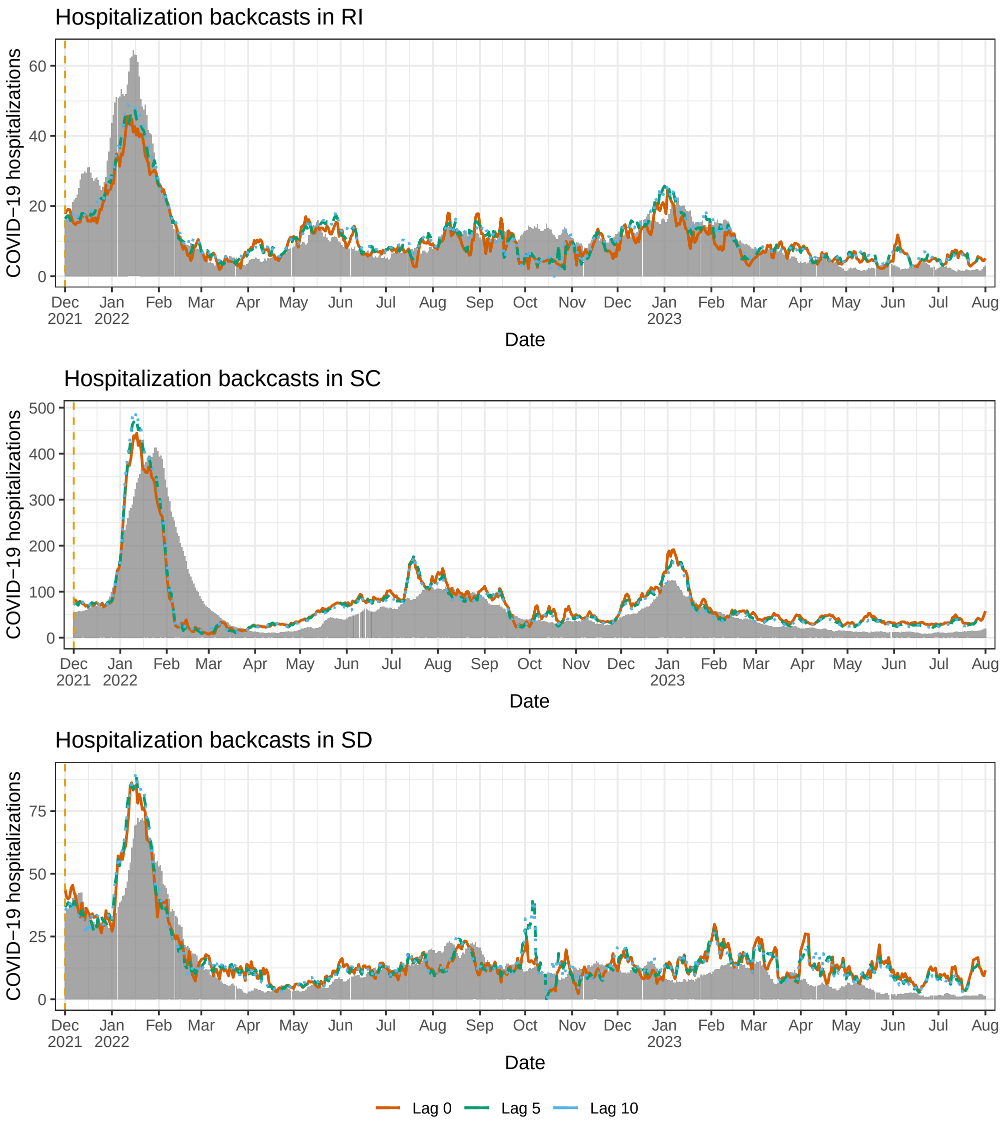

Fig S35. Backcasts from the mixed model in scenario 2, for RI, SC, SD.

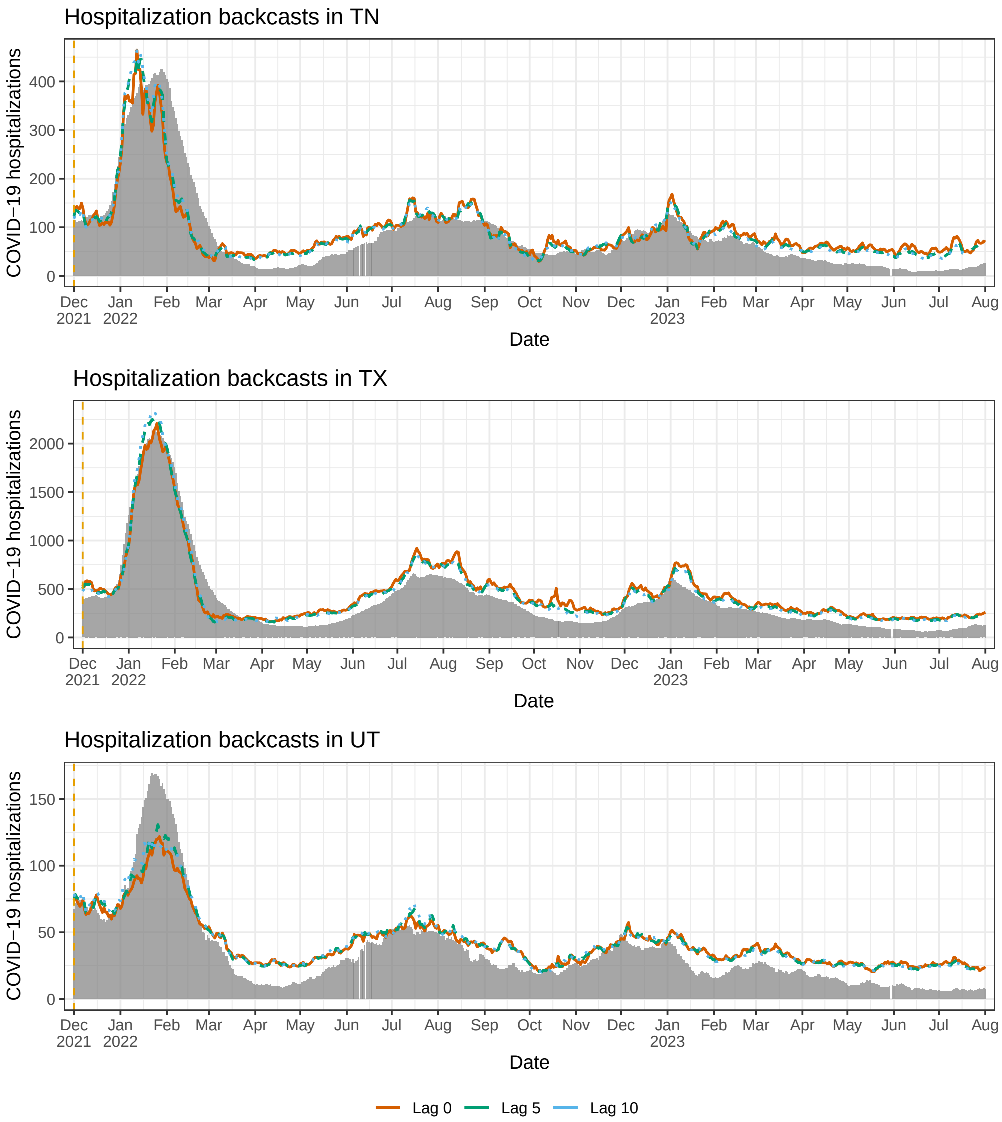

Fig S36. Backcasts from the mixed model in scenario 2, for TN, TX, UT.

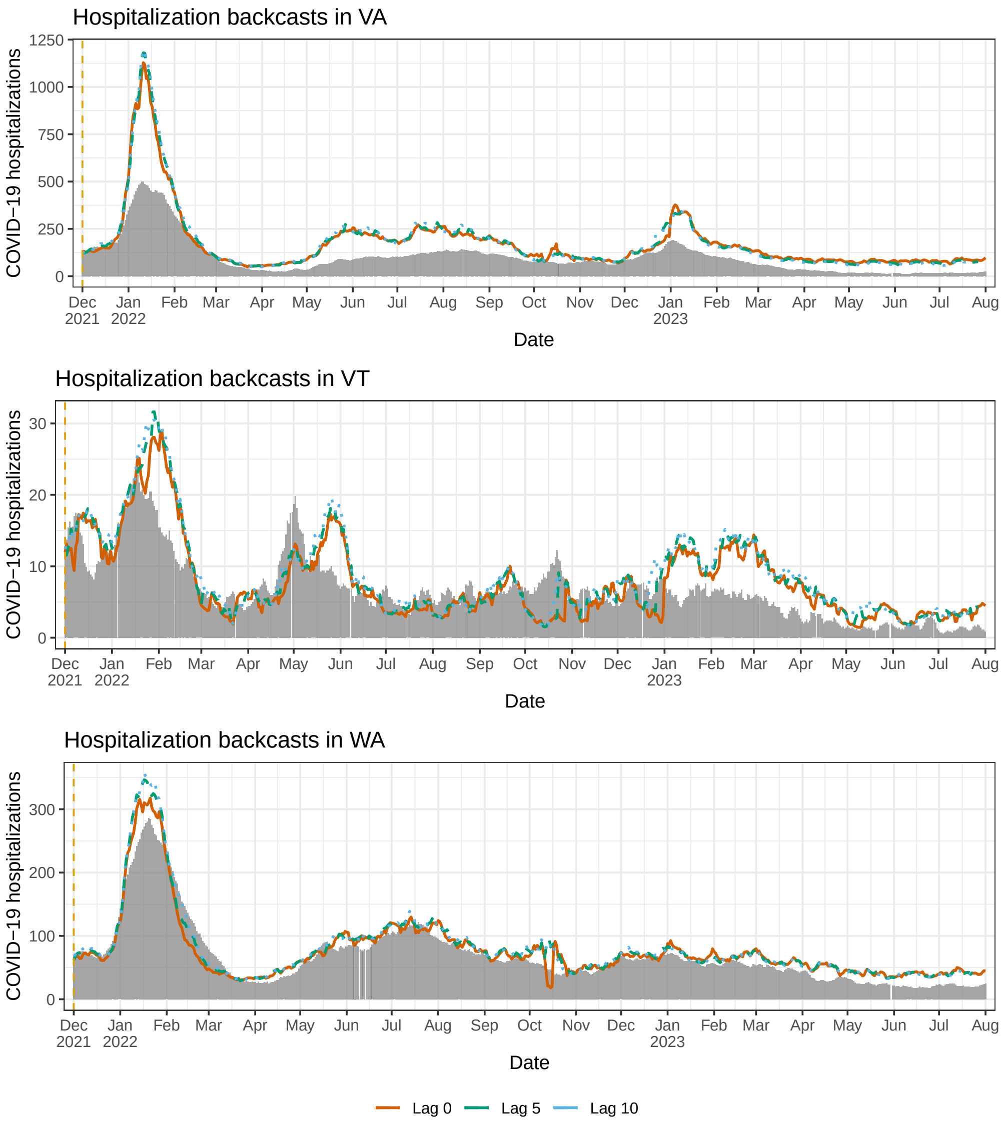

Fig S37. Backcasts from the mixed model in scenario 2, for VA, VT, WA.

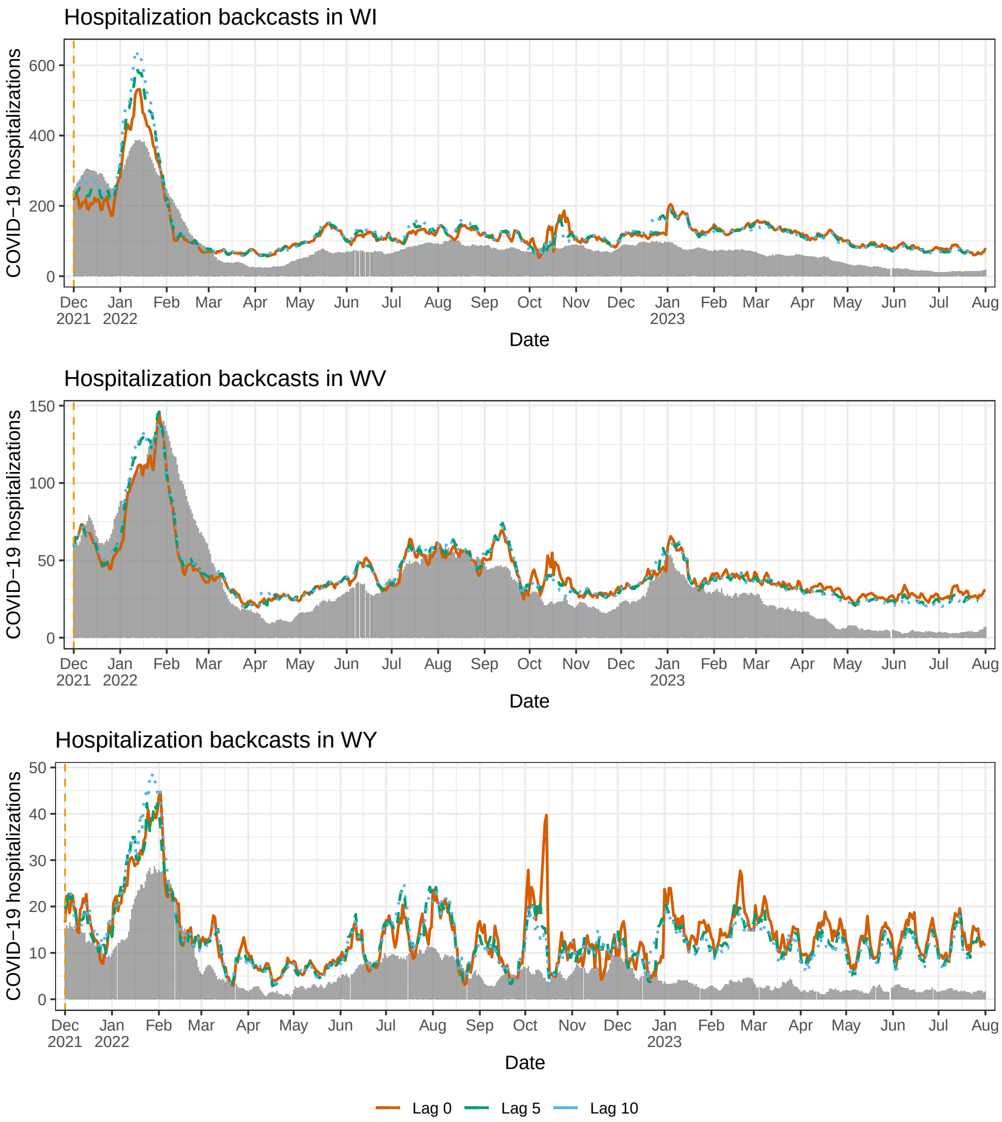

Fig S38. Backcasts from the mixed model in scenario 2, for WI, WV, WY.
